# Aptamers Chemistry: Chemical Modifications and Conjugation Strategies

**DOI:** 10.3390/molecules25010003

**Published:** 2019-12-18

**Authors:** Fadwa Odeh, Hamdi Nsairat, Walhan Alshaer, Mohammad A. Ismail, Ezaldeen Esawi, Baraa Qaqish, Abeer Al Bawab, Said I. Ismail

**Affiliations:** 1 Faculty of Science, The University of Jordan, Amman 11942, Jordan; f.odeh@ju.edu.jo (F.O.); hammdi2000@yahoo.com (H.N.); drabeer@ju.edu.jo (A.A.B.); 2 Hamdi Mango Center for Scientific Research, The University of Jordan, Amman 11942, Jordan; 3 Cell Therapy Center, The University of Jordan, Amman 11942, Jordan; 4 Faculty of Medicine, The University of Jordan, Amman 11942, Jordan; mohd.ismail2@yahoo.com (M.A.I.); ezaldeenesawi@gmail.com (E.E.); bqaqish@gmail.com (B.Q.); saismail@qf.org.qa (S.I.I.); 5 Qatar Genome Project, Qatar Foundation, Doha 5825, Qatar

**Keywords:** aptamers, drug delivery, nanocarriers, chemical modifications, conjugation

## Abstract

Soon after they were first described in 1990, aptamers were largely recognized as a new class of biological ligands that can rival antibodies in various analytical, diagnostic, and therapeutic applications. Aptamers are short single-stranded RNA or DNA oligonucleotides capable of folding into complex 3D structures, enabling them to bind to a large variety of targets ranging from small ions to an entire organism. Their high binding specificity and affinity make them comparable to antibodies, but they are superior regarding a longer shelf life, simple production and chemical modification, in addition to low toxicity and immunogenicity. In the past three decades, aptamers have been used in a plethora of therapeutics and drug delivery systems that involve innovative delivery mechanisms and carrying various types of drug cargos. However, the successful translation of aptamer research from bench to bedside has been challenged by several limitations that slow down the realization of promising aptamer applications as therapeutics at the clinical level. The main limitations include the susceptibility to degradation by nucleases, fast renal clearance, low thermal stability, and the limited functional group diversity. The solution to overcome such limitations lies in the chemistry of aptamers. The current review will focus on the recent arts of aptamer chemistry that have been evolved to refine the pharmacological properties of aptamers. Moreover, this review will analyze the advantages and disadvantages of such chemical modifications and how they impact the pharmacological properties of aptamers. Finally, this review will summarize the conjugation strategies of aptamers to nanocarriers for developing targeted drug delivery systems.

## 1. Introduction

Aptamers are single-stranded RNA or DNA oligonucleotides that fold up into a distinctive 3D structure capable of binding with high affinity and specificity to small molecules up to entire organisms, with nanomolar range dissociation constants. Nucleic acid-based aptamers were first described in 1990, where the first aptamers were in vitro selected using a random library of single-stranded oligonucleotides sequences [1] by a selection procedure known as systematic evolution of ligands by exponential enrichment (SELEX). The first oligonucleotide aptamer was isolated to bind with small molecules [2]. Nowadays, the aptamer field covers various biomedical applications, including [3], therapeutic [4,5,6], aptasensors [7], biosensors [8,9], diagnostic [10,11], and imaging systems [12].

Aptamers need to be stabilized for in vivo use against nuclease degradation, and their small size makes them susceptible to renal filtration. Aptamers’ stabilization can be attained by chemically modifying them using different approaches. Moreover, introducing chemical modifications into nucleic acid libraries increases the interaction capabilities of aptamers and thereby their target spectrum [12]. Modified aptamers may show improved chemical diversity relative to aptamers composed entirely of natural DNA or RNA nucleotides and expand their applications in diagnostics, therapeutics, and nanotechnology [1].

Chemical modifications of aptamer oligonucleotides are needed mainly to enhance their resistance to nuclease degradation and lowering their renal filtration. Additionally, chemical modifications, in some cases, may increase the aptamer-binding affinity [13]. Many approaches have been introduced to promote the stability of aptamers without altering their binding affinity and specificity against their targets. These approaches include chemical modification of the phosphate backbone [14,15], oxygen replacement with sulfur on the ribose unit and into phosphodiester linkage, end capping at the 3′ and/or 5′ terminals [16,17], locked nucleic acids [18,19], and circular [20], multivalent, and dimerization of aptamers [21,22]. Modification of the 2′-position of the ribose sugar is the most common [23,24].

Other aptamer chemical modification strategies were also required for conjugating aptamers to different drug molecules [25] or for active targeting nanoparticles [26]. These strategies, usually, introduce an active functionality either at the 3′ or 5′ terminals of the oligonucleotide to interact with their coupling partner on the surface of nanoparticles. Several coupling chemistry methods have been applied for aptamer conjugation, including thiol-maleimide [27], carbodiimide [28], oxidative coupling [29], thiol–gold coordination [30], avidin–biotin coupling [31], and click chemistry [32].

## 2. Aptamers and Selection Methods

Aptamer target-binding specificity and affinity are obtained using a Darwinian evolution screening technology called systematic evolution of ligands by exponential enrichment (SELEX). The SELEX methodology is quite similar for both DNA and RNA aptamers. However, RNA aptamers require an additional step of reverse transcription before amplification. Targets on which aptamer selection is conducted against are diverse, ranging from ions to whole living cells. SELEX starts by chemical synthesis of a library of random sequences of double-stranded DNA (dsDNA). The dsDNA library is either used to synthesize a single-stranded DNA (ssDNA) library or undergoes in vitro transcription to produce an ssRNA library. The resulting library sequences have the ability to fold, forming unique 3D structures. Conditions of selection, such as temperature, pH, and ionic strength, can be controlled to be compatible with the application of interest. Conventional SELEX generates aptamers by first incubating a library composed of 10^13^ to 10^15^ different folded oligonucleotide sequences with the target of choice. This library is composed of random sequences in the middle and constant regions at the 5′- and -3′ ends, which are used for primer annealing and amplification. After incubation, the bound sequences can be separated from the remaining unbound sequences, retained, and then amplified to be reintroduced in iterative selection cycles. Conditions can be changed through each cycle to achieve optimal aptamer target-binding affinity. Rounds of selection are repeated until optimal enrichment for the highest affinity and specificity aptamers are achieved. Approximately 20 rounds of selection would yield high target affinity aptamers [33].

Since the discovery of aptamers, a number of SELEX methods have been developed, as demonstrated in Table 1. One important variant of SELEX is the cell-based selection methodology (cell-SELEX). Cell-SELEX proved effective in developing aptamers for targets in their native status, biomarker discovery, and pathogen-infected cells. Cell-SELEX also gives an added benefit of selecting aptamers against targets existing in their original cells, which increases the specificity of selected aptamers. In cell-SELEX, the bound sequences are detached from target cells, amplified, and reintroduced to target cells in subsequent rounds of enrichment while unbound sequences are washed out. Specificity against target cells can be achieved through an extra step of counter selection, where the library is tested against control cells that are related to the target cells.

Another SELEX variant is in vivo SELEX. This method generates aptamers against targets in living organisms instead of using isolated pure targets or individual cells. This method is useful in overcoming some of the challenges facing aptamers selected by in vitro SELEX methods. The principle here is similar to that of conventional SELEX and cell SELEX. However, the library of aptamers can be injected into the peripheral vasculature of the living organism followed by tracking and isolation of specific aptamers homed into target tissues. In Vivo SELEX has been successfully applied for different diseases, including cancers and viral-infected cells [45,46,47].

Aptamers have many superior advantages over antibodies as the targeting ligand, including shorter production time, lower synthesis costs, better thermal stability, and a wider spectrum of targets. Additional main comparisons and differences are summarized in Table 2.

## 3. Chemical Modifications of Aptamers and Their Impact on Pharmacological Properties

Despite the numerous encouraging characteristics of aptamers, they bear several drawbacks [52], such as (i) decreased biostability mainly due to rapid renal excretion and nuclease hydrolysis, (ii) nucleic acids lack functional groups that could enhance the binding affinity through extra potential interactions, and the (iii) intra-nucleotide chemical modification of aptamers dramatically affects the binding affinity. 

In order to overcome these problems, modifications located at the sugar unit, the nucleobase, or the backbone of the constituting nucleotides can be introduced to enhance aptamer biostability and binding affinity [53]. Aptamer modification can be achieved either into the scaffold of selected aptamers through standard solid-phase synthesis (post-selection modification) or by using modified nucleoside triphosphates (NTPs) directly in the selection process [54,55,56]. The common chemical modification approaches of nucleic acid aptamers for the development of clinical therapeutics are discussed in the following sections.

### 3.1. Modifications on Nucleic Acids Terminals

#### 3.1.1. Terminal 3′–3′ and/or 5′–5′ Internucleotide, 3′ and 5′-Biotin Conjugates

Capping the 3′-end is one of the generally used strategies to block 3′ to 5′ exonuclease attack. Capping the 3′-end with inverted deoxy-thymidine modification is usually used to increase the stability and resistance of aptamers against 3′-exonuclease in human serum [57]. This modification needs a modified control pore glass (CPG) with the 5′-hydroxyl of the first nucleoside attached, followed by chain elongation using the classic solid-phase phosphoramidite process [58].

**Figure 1:**
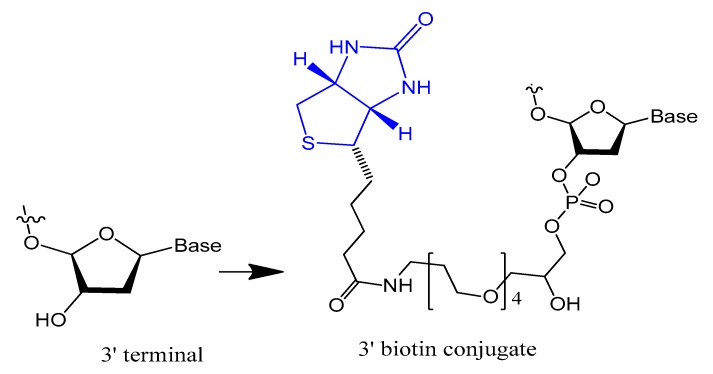
Structure of the 3′-biotin conjugate.

The 3′-biotin aptamer modification is also reported to enhance stability against 3′-exonuclease (Figure 1) [57]. The 3′-biotin-streptavidin attached to thrombin aptamer was investigated against 3′-exonuclease activity in the blood of rodents. The results showed that 3′-biotin modification significantly enhances 3′-exonuclease resistance and decreases the clearance rate of aptamers in the blood circulation in vivo (10- to 20-fold) [16]. The 3′-biotin modified-DNA aptamer targeting severe acute respiratory syndrome (SARS) coronavirus helicase was sustained in fetal bovine serum (FBS) in double the time compared to unmodified aptamer [57].

Moreover, the stability of selected non G-quadruplex aptamer (NG8) that was modified with 3′-biotin or 3′-inverted thymidine was increased. The 3′-biotin and 3′-inverted thymidine NG8 aptamer was strongly resistant to nuclease attack in serum compared to the unmodified NG8 aptamer. The 3′-inverted thymidine aptamer remained intact for up to 72 and 31 h in 5% and 10% FBS, respectively. The 3′-inverted thymidine modification showed higher stability than the 3′-biotin modification, but both modifications were significantly more stable than the unmodified aptamer [57].

Riccardi et al. synthesized and characterized a dansyl-fluorescent thrombin-binding aptamer (TBA) analog, named tris-mTBA, functionalized with a 5′-biotin tag, for incorporation onto streptavidin-coated silica NPs. The results showed that tris-mTBAwas able to form an antiparallel G-quadruplex structure and retain the ability to form a duplex structure with its complementary strand (cTBA), which acts as an antidote to reverse the anticoagulation activity of TBA. Moreover, they proved that tris-mTBA inhibits the human thrombin activity 10-fold more efficiently than unmodified TBA and biotin-TBA and in a reversible manner. In addition, TBA analogs showed higher resistance to enzymatic degradation compared to the unmodified TBA due to a protective effect of the conjugating groups [59].

Ortigao et al. found that a minor structural change of an oligonucleotide at the two terminal internucleotide bonds, a 3′,3′ and a 5′,5′ linkage, is sufficient to stabilize these end-inverted (INV) oligonucleotides against nuclease degradation. INV oligonucleotides are degraded very slowly in biological systems, in human serum with a half-life of ~36 h, and in *Xenopuslaevis* oocytes with a half-life of ~10 h, whereas control "normal" oligonucleotides are completely degraded in less than 30 min in both systems [60].

#### 3.1.2. 5′-End with Cholesterol and Other Lipid Moieties

Small aptamers are cleared and excreted rapidly by renal glomerular filtration. To overcome renal filtration and extend the circulation period, aptamer modifications with hydrophobic and/or bulky moiety are required [61,62]. Cholesterol was conjugated at the 5′-end of a 16-mer oligonucleotide (ODN) through a phosphate spacer, then incubated with low-density lipoprotein (LDL), leading to the formation of a cholODN-LDL. The plasma half-life of the cholODN-LDL aptamer was nearly 10 times better than the plasma half-life of the unmodified aptamer. Furthermore, the modified cholODN-LDL version showed high stability against rat serum nucleases [17].

Recently, a cholesterol-conjugated and 2′-F pyrimidine-modified RNA aptamer targeting the hepatitis C virus (HCV) NS5B protein was modified by Lee et al. This aptamer modification extended the aptamer plasma circulation time nine-fold compared to the unmodified version and enhanced the aptamer exposure to its target [63]. In another case, a 5′-cholesterol-modified oligonucleotide (ARC155) showed more rapid plasma clearance relative to the unconjugated aptamer, which was explained by the inability of the ARC155 folded structure to bind with plasma lipoproteins as other cholesterol-attached aptamers [61].

**Figure 2:**
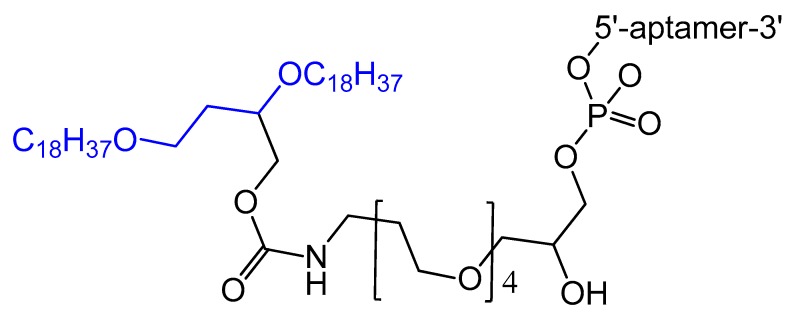
Synthesis of the diacylglycerol (DAG)-modified VEGF aptamer.

A diacylglycerol (DAG) lipid anchor was conjugated to the 5′-end of vascular endothelial growth factor (VEGF) aptamer (Figure 2). This 5′-end DAG-modified VEGF aptamer was incorporated into the bilayers of liposomes, which resulted in aptamers with improved inhibitory activity toward VEGF-induced endothelial cell proliferation in vitro and increased vascular permeability in vivo. Moreover, the residence time in plasma was considerably improved when compared to that of free aptamers [64].

**Figure 3:**
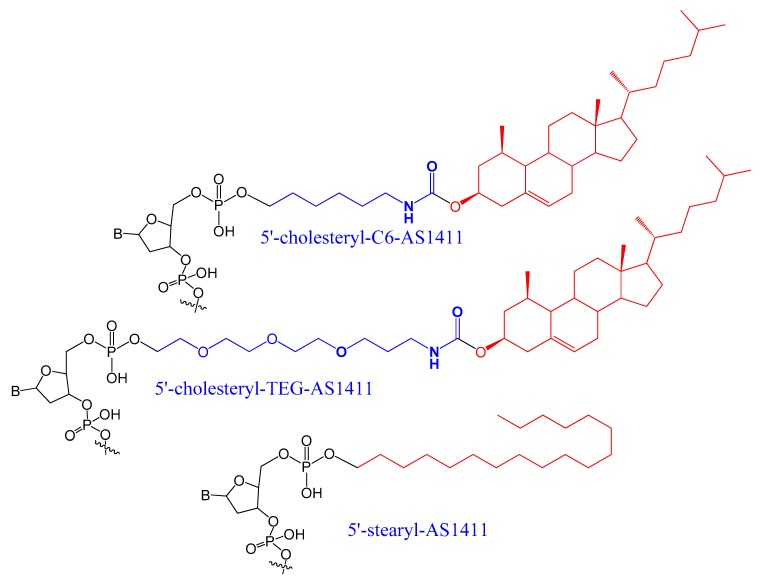
A set of lipids conjugated to 5′-AS1411 aptamer (stearyl- or cholesteryl-based tails.

A set of lipids conjugated to 5′-AS1411 aptamer (stearyl- or cholesteryl-based tails) (Figure 3) were selected and investigated for their conformational behavior and aggregation tendency in comparison with unmodified AS1411. The 5′-lipidated AS1411 derivatives folded into stable unimolecular G-quadruplex structures, forming large aggregates at a concentration of higher than 10 μM, and they maintained a similar biological behavior as unmodified aptamer with less cytotoxicity on the selected three different cancer cell lines [65].

#### 3.1.3. 5′-End PEGylation

The conjugation of polyethylene glycol (PEG) to drugs has been shown to increase the residence time of the drug in the body and decrease degradation by metabolic enzymes. PEG is non-toxic and nonimmunogenic and is approved by the Food and Drug Administration (FDA) [66].

**Figure 4:**
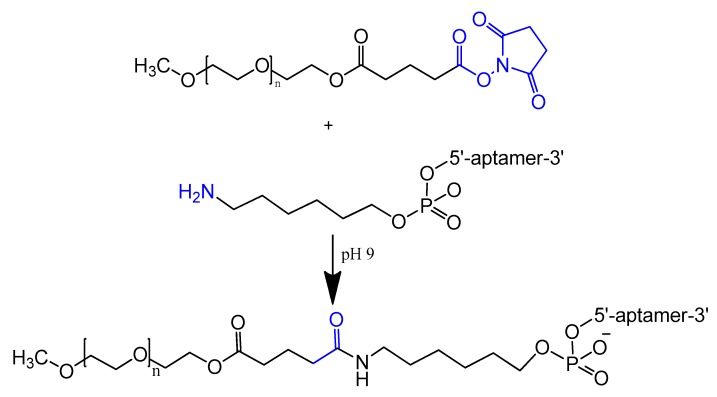
Reaction scheme of aptamer conjugation to a 40-kDa polyethylene glycol (PEG) at the 5′ terminal.

An amino-modified spiegelmer NOX-E36 oligonucleotide was conjugated with (NHS)-ester-activated polyethylene glycol via carbodiimide coupling. This combination formula with high molecular weight PEG had the advantages of both nuclease resistance and decreased renal excretion [67]. MP7 is a DNA aptamer that binds to the murine extracellular domain of PD-1 (programmed death protein 1). Conjugation of MP7 DNA aptamers with large PEG molecules at the 5′ terminal via carbodiimide chemistry (Figure 4) could limit the rate of filtration and extend the half-life of this small molecule up to 24 to 48 h [62].

An interesting new PEGylation method, sbC-PEGylation, was introduced recently for RNA aptamers acting against interleukin-17A (IL-17A) in mice and monkeys. These sbC-PEGylated aptamers were synthesized by coupling the symmetrical branching molecule 2-cyanoethyl-*N*,*N*-diisopropyl phosphoramidite to the 5′ end of the aptamer, before conjugating two PEG molecules to the aptamer. The sbC-PEGylated aptamers had improved pharmacokinetic properties and showed excellent stability in the blood circulation. Moreover, one of the sbC-PEGylated aptamers, 17M-382, inhibited interleukin-6 (IL-6) in a concentration-dependent manner, with the IC_50_ of 17M-382 two times lower than that of non-PEGylated 17M-382 [68].

The bifunctionalized anti-MUC1 aptamer (NH_(2)_-AptA-SR) was conjugated with different PEG types (either a conventional branched PEG or the comb-shaped polyPEG) to enhance its biodistribution properties against MCF-7 cell lines. The body clearance data showed that more than 60% of the un-PEGylated aptamer was excreted after 5 h compared to 43% to 51% in the case of the newly modified aptamers [69].

The PEGylation of doxorubicin-attached anti-MUC1 aptamer (PEG-APT-DOX) increases the targeting efficacy of the aptamer by reducing the non-specific uptake of doxorubicin by RAW 264.7 macrophages. PEG-APT-DOX kept more than 80% of the RAW cells viable while killing more than 60% of the MCF7 cells. This proves the desirable cytotoxic effect of doxorubicin to MCF7 cells was not hindered by the modification [70].

### 3.2. Modifications on the Sugar Ring

#### 3.2.1. 2′-Substitutions

**Figure 5:**
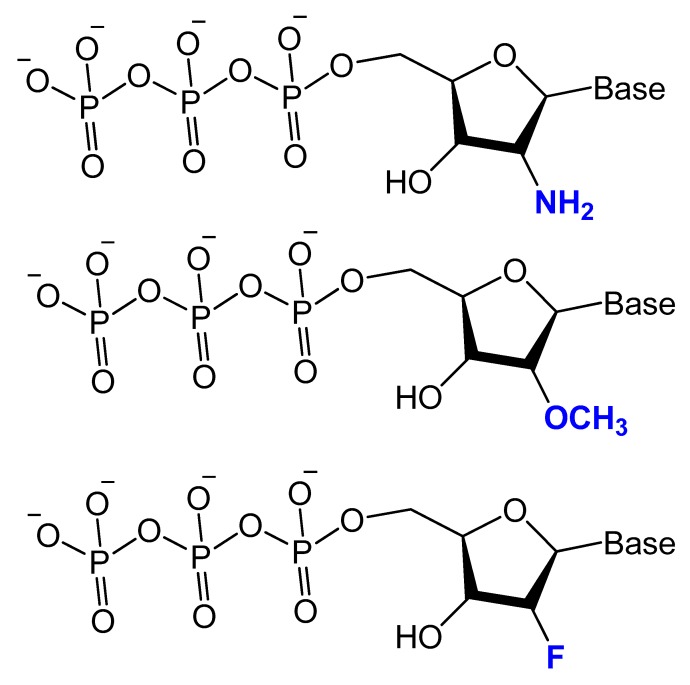
Chemical structures of 2′-modified nucleotides used in selection experiments to generate aptamers with enhanced pharmacokinetic properties.

Modifications on the 2′ position of the sugars were effective at improving the aptamer serum half-life. The 2′-Fluoro (2′-F), 2′-amino (2′-NH_2_) and 2′-*O*-methyl (2′-OMe) are the most common 2′-substitute modifications on the ribose unit. Usually, it is used to increase nuclease resistance and optimize aptamer affinity (Figure 5) [23,24,71].

For example, two 2′-F-modified thrombin-binding aptamers (PG13 and PG14) showed approximately a four-fold increased binding affinity to thrombin and up to seven-fold higher nuclease resistance. The G-quadruplex stability of the modified aptamer was increased up to 48-fold in 10% FBS [13]. Lin et al. developed a 2′-NH_2_ group-modified RNA aptamer against human neutrophil elastase (HNE). This modified aptamer showed a good binding affinity and 10-fold enhanced stability in human serum and urine compared to unmodified aptamer [72]. High-affinity 2′-amino-2′-deoxypyrimidine-modified RNA ligands have been reported to be potent inhibitors of basic fibroblast growth factor (bFGF). Compared to unmodified RNA with the same sequence, 2′-aminopyrimidine ligands are at least 1000-fold more stable in 90% human serum [55]. 2′-Amino-modified RNA and DNA aptamers that bind to vascular permeability factor/vascular endothelial growth factor (VPF/VEGF) have been investigated by Green et al. They showed nuclease resistant properties with high binding affinity [73].

Pagratis et al. demonstrated, by in vitro selection-amplification from random libraries of RNA molecules containing 2′-NH_2_ or 2′-F-2′-deoxypyrimidines, that 2′-F RNA ligands have higher binding affinities (K_D_ ranging between 0.3 and 3 pM) for 2′-F compared to 400 pM for 2′-NH_2_, and bioactivities with extreme thermo-stabilities compared to 2′-NH_2_ ligands [74]. Pegaptanib is a 2′-multimodified RNA aptamer, and it binds with very high affinity to the human vascular endothelial growth factor for the treatment of neovascular age-related macular degeneration (AMD). It is an example of an aptamer-based drug currently approved by the FDA [75]. Maio et al. designed a 2′-*O*-methyl RNA analog to a DNA aptamer, resulting in increased aptamer stability towards nucleases for up to 24 h without affecting affinity against myeloid leukemia [24]. In addition, anti-VEGF combined the use of 2′-fluoro and 2′-methyl pyrimidine NTPs and wild-type purine NTPs followed by the post-selection modification of the unmodified ribonucleotides with 2′-methoxy modifications. These aptamers bind equally well to murine VEGF164, do not bind to VEGF121 or the smaller isoform of placenta growth factor (PlGF129), and show a reduced but significant affinity for the VEGF165/PlGF129 heterodimer modifications [71].

Li et al. selected and isolated a nuclease-stable 2′-fluoropyrimidine-modified anti-EGFR aptamer to target four members of the epidermal growth factor receptor (EGFR) family. A promising aptamer (E07) had a strong binding affinity (K_D_ = 2.4 nM) for the wild-type receptor, leading to cell proliferation inhibition [76]. Esposito et al. isolated 2′-fluoropyrimidine-modified anti-EGFR that showed a high binding affinity to the lung cancer cell line, A549 [77]. A 2′-fluoro-modified RNA aptamer (S2) was generated against the prostate-specific antigen (PSA). The modified aptamer, being highly stable in human serum, showed a modest affinity (K_D_ = 630 nM) to PSA [78]. In addition, other 2′-F-RNA aptamers (A10) had been isolated and tested for their potential treatment of human prostate cancer cells via the prostate-specific membrane antigen with a K_D_ of 11.9 nM [79]. Other interesting aptamer is A15 aptamer, a 2′-fluoropyrimidine modified RNA aptamer, this aptamer was isolated from the brain of mice by in vivo SELEX after mice tail injection with 2′-fluoro-modified RNA library. The isolated A15 aptamer was further modified with 2′-methoxy residues to increase the nuclease resistance and tested for brain penetration. The biodistribution of the A15 aptamer verified positive signals in different brain regions [47].

Recently, Thirunavukarasu et al. reported the selection of two types of 2′-F purine aptamers (2fHNE-1 and 2fHNE-2) that bind human neutrophil elastase (HNE). They bind HNE with reasonable affinity and the 2′-F purines substituent enhanced nuclease resistance [80].

Despite 2′-methoxy modification being one of the post-selection procedures, due to bulky 2′-OMe-NTPs [81], a combination of three 2′-OMe-NTPs was used in a SELEX experiment to generate an aptamer that binds to the polypeptide tissue factor pathway inhibitor (TFPI). This modified aptamer showed high selectivity and binding affinity to correct thrombin generation hemophilia A and B [82].

These 2′-modified aptamers can be easily conjugated, as unmodified aptamers, to different nanocarriers loaded with certain chemotherapeutic agents. For example, 2′-OMe-modified aptamers conjugated to a polymeric nanoparticles loaded with docetaxel, an anticancer agent, showed a high specificity as well as a targeted toxicity improvement [83].

A 2′-deoxy-2′-fluoro-modified arabinonucleotide (2′F-ANA) was investigated based on a thrombin-binding DNA aptamer d(GGTTGGTGTGGTTGG), an anti-HIV phosphorothioate PS-d(TTGGGGTT), and a DNA telomeric sequence d(GGGGTTTTGGGG) by UV thermal denaturation (Tm) and circular dichroism (CD) experiments. The results showed that the replacement of deoxyguanosines that adopt the anti-conformation (antiguanines) with 2′F-arabino guanosines can stabilize G-quartets and maintain the quadruplex conformation while replacement of syn-guanines with 2′F-arabino guanosines is not favored and results in a dramatic switch to an alternative quadruplex conformation. In addition, the data showed that the appropriate incorporation of 2′F-ANA residues into G-quadruplexes leads to an increase in the melting temperature of the complex formed. Moreover, the nuclease resistance of 2′F-ANA-modified thrombin-binding aptamers was increased up to 48-fold in 10% FBS, with a 4- to 5-fold enhancement in binding affinity to thrombin [13]. Similarly, Wilds and Damha used UV thermal melting and CD experiments to discover the thermodynamic stability and helical conformation of 2′F-ANA/RNA and 2′F-ANA/DNA hybrids. They showed that 2′F-ANA had enhanced RNA affinity to RNase H enzyme relative to that of DNA and phosphorothioate DNA. The 2′F-ANA modification also showed favorable pairing to single-stranded DNA [84].

The stability of 2′-deoxy-2′-fluoroarabinonucleic acid (2′F-ANA) to hydrolysis has been investigated under acidic and basic conditions. 2′F-ANA was found to have increased stability compared to both DNA and RNA in enzyme-free simulated gastric fluid (pH ~1.2). Under basic conditions, 2′F-ANA also showed good stability. Furthermore, phosphorothioate-2′F-ANA linkage was found to be much more susceptible to enzymatic cleavage than the phosphorothioate-DNA [85].

The differential stability of 20 F-ANA RNA and ANA RNA hybrid duplexes was evaluated by NMR and theoretical calculations. An increased binding affinity of 2′ F-ANA was observed due to a favorable pseudo hydrogen bond (2′ F–purine H8), which contrasts with unfavorable 2′-OH–nucleobase steric interactions in the case of ANA. The 2′ F-ANA strand′s structure was more compatible with the A-like structure of a hybrid duplex and more suitably reorganized for duplex formation [86].

#### 3.2.2. 4′-Oxygen Replacement

**Figure 6:**
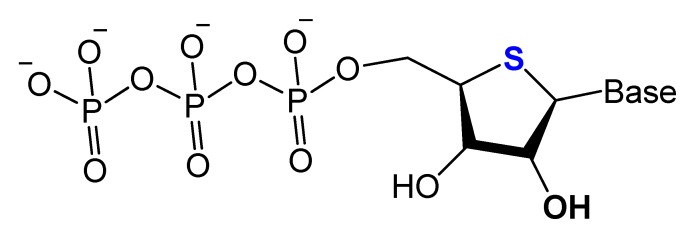
Structure of 4′-thioNTPs.

Replacing the 4′-oxygen atom of the sugar unit with a sulfur atom is rarely utilized in selection experiments of aptamer isolation (Figure 6) [87]. Synthesized 4′-thiouridine (4′-thio-UTP) and 4′-thiocytidine (4′-thio-CTP) triphosphates were used in the in vitro selection of anti-thrombin thioRNA aptamers. The 4′-thio-modified aptamer showed a high affinity (K_D_ = 4.7 nM), with a 50-fold increase in resistance to RNase A [88]. Minakawa et al. isolated fully modified 4′-thioRNA aptamers against human alpha-thrombin using four types of 4′-thioribonucleoside triphosphates (4′-thioNTPs). The modified aptamer displayed a similar binding affinity to thrombin as the partially modified aptamer (K_D_ = 7.2 nM) [87].

#### 3.2.3. Locked and Unlocked Nucleic Acid

**Figure 7:**
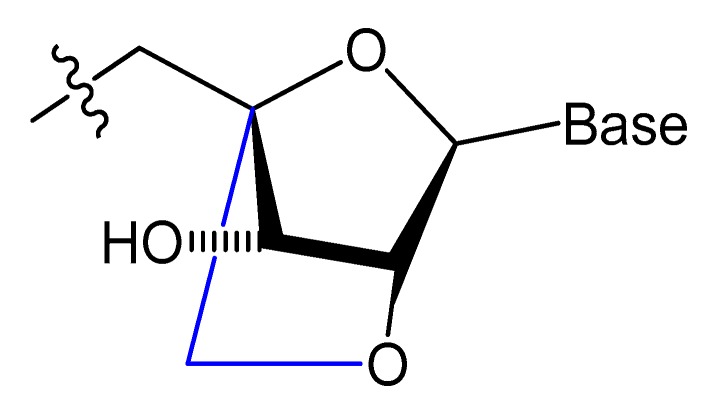
Structure of LNA monomers [19].

A methylene linkage between 2′-*O* and 4′-*C* of the sugar ring produces an analog of ribonucleotide called locked nucleic acid (LNA) (Figure 7). This modification showed a better thermostability and vastly enhanced nuclease resistance [18,19].

**Figure 8:**
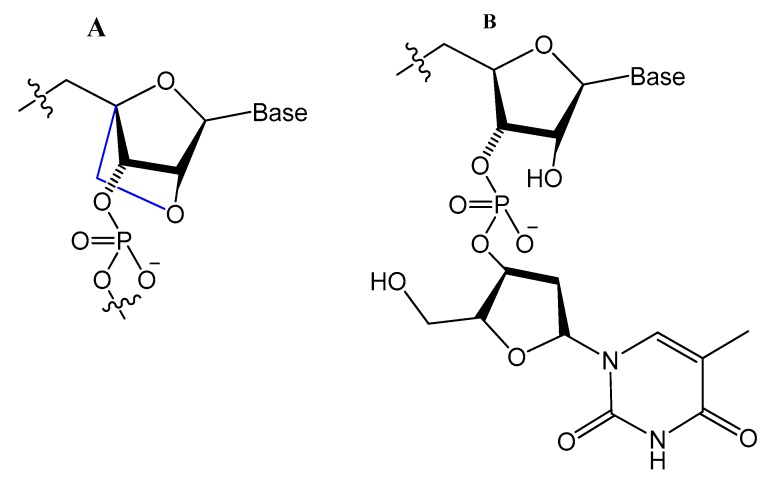
(**A**) The structure of LNA, a derivative of ribonucleotide with a methylene bridge. (**B**) The structure of 3′-3′-T, a common approach to block the 3′-exonuclease attack [90].

LNA/DNA chimera LNA5, forming a stable complex against HIV-1 trans-activating response (TAR) RNA, was synthesized from a shortened and stable version of the hairpin RNA aptamer identified by in vitro selection against TAR. The results indicated that these modifications provide good protection towards nuclease digestion in bovine serum and keep the same binding affinity of the unmodified RNA aptamer [89]. Shi et al. developed another LNA/DNA chimeric aptamer probe through proper LNA incorporation and 3′-3′-thymidine (3′-3′-T) capping (Figure 8). The serum stability of the modified TD05 aptamer, a DNA aptamer against lymphoma Ramos cells, was gradually enhanced by 10-fold, with maintained affinity and specificity to Ramos cells [90].

**Figure 9:**
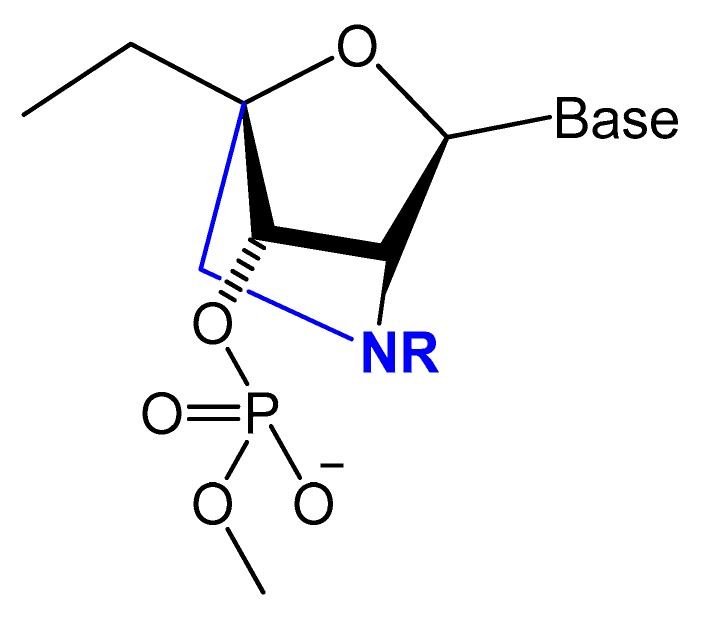
Structures of LNA and 2′-amino-LNA nucleotide monomers.

A Tenascin-C-binding aptamer was modified with LNA nucleotides (TTA1) that exhibited improved plasma stability and maintained strong binding to Tenascin-C [91]. Moreover, an avidin-binding DNA aptamer was modified systematically with LNA and a 2′-amino derivative as demonstrated in Figure 9. At certain positions, the modified aptamer actually showed improved binding affinity (K_D_ value of 4.20 nM) [92].

**Figure 10:**
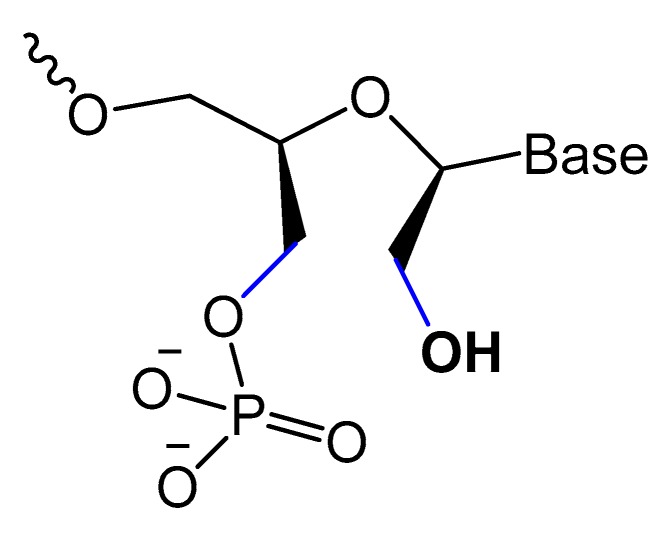
Structures and furanose conformation of UNA nucleotide [12].

Unlocked nucleic acid (UNA) is another modification in the ribose unit, achieved by eliminating the single bond between C2′ and C3′ of the sugar. Such a modification makes the aptamer more flexible. This structure flexibility may ease the strain in tight aptamer loops (Figure 10) [12]. The 15-mer thrombin-targeted DNA that underwent UNA modifications on the loop regions showed increased thermodynamic stability, with significant aptamer affinity and anticoagulant efficiency [93].

A thrombin-binding quadruplex aptamer with a UNA-modified thrombin-binding aptamer (UNA-modified TBA) was developed. It was found that UNA substitution in the loops of the quadruplex could increase the binding affinity and clotting time in blood samples. The UNA monomer is allowed in many positions of the aptamer without significantly changing the thrombin-binding properties [93].

Aptamers could be selected with LNA or UNA in their structure, which may give better results. Three different LNA-nucleoside triphosphates, LNA-TTP, LNA-ATP, and LNA-5-methyl-CTP, were tested as substrates for KOD DNA polymerase. The results showed that KOD DNA polymerase is good for the synthesis of DNA oligonucleotide duplexes containing LNA nucleotides [94].

The effect of the presence of nucleosides in unlocked nucleic acid (UNA), locked nucleic acid (LNA), or β-l-RNA series, as analogs to RE31-DNA aptamer for effective prolonging of the thrombin time, was evaluated by Kotkowiak et al. They showed that all modified residues can influence the thermal and biological stabilities of G-quadruplex in a position-dependent manner. The aptamers modified simultaneously with UNA and LNAs possess a two-fold higher anticoagulant effect. RE31 variants modified with nucleosides in UNA, LNA, or β-l-RNA series exhibited prolongation of aptamer stability in human serum [95].

### 3.3. Modifications on the Phosphodiester Linkage

#### 3.3.1. Methylphosphonate or Phosphorothioate

**Figure 11:**
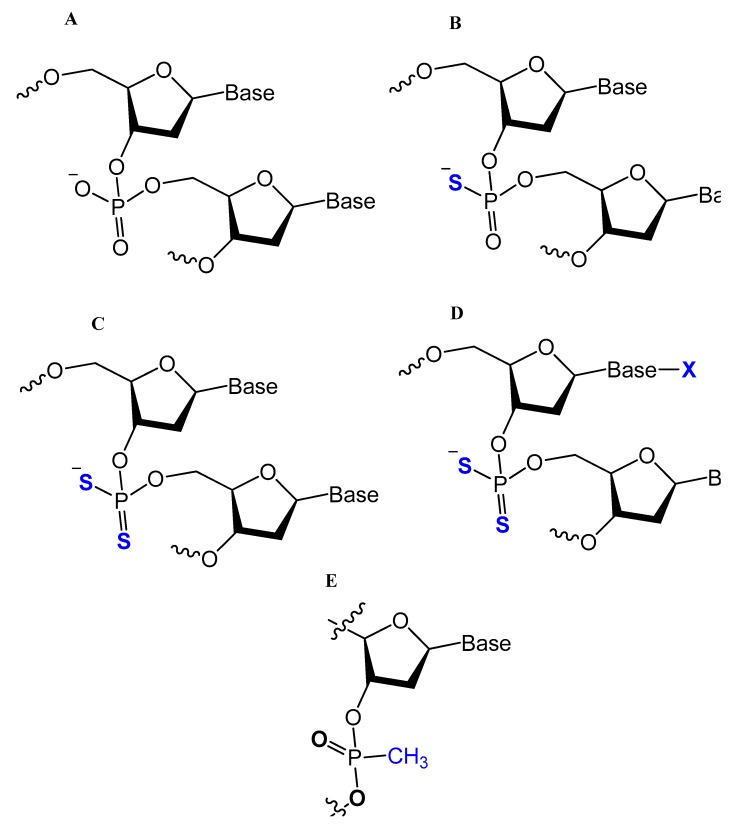
Chemical structures of modified oligonucleotides: (**A**) Normal, phosphodiester backbone; (**B**) Mono-thio-modified thioaptamer; (**C**) Di-thio-modified thioaptamers. (**D**) Di-thio-modified X-aptamer; and (**E**) methylphosphonate [50].

One of the common aptamer modifications can be achieved by replacing the phosphodiester linkage with methylphosphonate or phosphorothioate on the α-phosphorous (Figure 11) [14,52].

The phosphorothioate modification might influence the thermal stability of the quadruplex structure in different G-quadruplex-forming oligonucleotides, and the phosphorothioate backbone is considered to be responsible for strongly binding and inhibiting the gp120 envelope protein of the HIV [14]. Partial thiophosphorylated substitutions with maximum thermal stability were selected for evaluating their stabilities under conditions of nuclease RQ1 DNAse hydrolysis and their antithrombin activities in blood plasma. A promising modified oligonucleotide (GGTTGGTGTGGTTGG), with the structure modified only in TT loops (LL11), retained thrombin-binding aptamer properties with high resistance to biodegradation [96]. A thrombin-binding aptamer, d(GGSTSTSGGTGTGGSTSTSGG), with thio-substitutions in both TT loops exhibited a similar antithrombin efficiency compared to the unmodified one, with better resistance to DNA nuclease in blood serum [15]. Abeydeera et al. reported that phosphorodithioate (PS2) substitution on a single nucleotide of RNA aptamers improved the target binding affinity by 1000-fold by stabilizing the phosphate backbone [97].

Post-SELEX modifications by the addition of short phosphorothioate caps to the 3′- and 5′-ends of the 2′-amino-modified RNA and DNA aptamers against VPF/VEGF showed high binding affinity and increased nuclease resistance [73].

Mann et al. developed a two-step selection protocol for identifying a thiophosphate-modified aptamer against E-selectin (ESTA-1). The isolated ESTA-1 aptamer showed nuclease resistance and specifically bound to E-selectin with high affinity (K_D_ = 47 nM) without recognizing the other members of the selectin protein family. ESTA-1 aptamer bound specifically to the inflamed tumor-associated vasculature of human carcinomas derived from breast, ovary, and skin without affecting normal organs [98].

Different types of phosphorothioate aptamers have been isolated via in vitro combinatorial selection. For example, King et al. selected purified recombinant human NF-kappa B proteins, RelA(p65) and p50, duplex thioaptamers. These phosphorothioate aptamers showed high affinity besides competitive binding with the duplex 22-mer-binding site, Ig kappa B [99].

Somasunderam et al. utilized the same protocol to isolate phosphorothioate aptamers acting as inhibitors of the RNase H domain of HIV-1 reverse transcriptase with high affinity (K_D_ of 70 nM) [100]. They also separated monothiophosphate-modified aptamers that specifically bind to CD44 with a high affinity in the range 180–295 nM, an affinity significantly higher than that of hyaluronic acid [101]. The selection of a thioaptamer targeting the Dengue virus type-2 envelope (DENV-2) protein domain III was also achieved by Gandham et al. DENTA-1 thioapatamer binds to DENV-2 EDIII, with a dissociation constant of 154 nM [102].

Recently, thiophosphate ester aptamers (TA), selected from large combinatorial libraries, with CD44 (CD44TA) targeting moiety, was attached to discoidal silicon mesoporous microparticles (SMP) to improve the accumulation of these carriers in infected macrophages in the lungs. This thioaptamer significantly lowered the bacterial load in the lungs, caused recruitment of T lymphocytes, and enhanced binding affinity and specificity for proteins as well as stability in vitro [103,104].

X-aptamers were the next generation of phosphorothioate aptamers, in which a C5 position of the nucleobase was modified with a drug-like functionality [105]. This modification showed a significant enhancement of nuclease resistance and increased binding affinities [106]. The best X-aptamer modification with small molecule drugs and antibodies can be achieved if these molecules can fold into unique 3-D structure scaffolds to bind specifically to the target protein [50].

**Figure 12:**
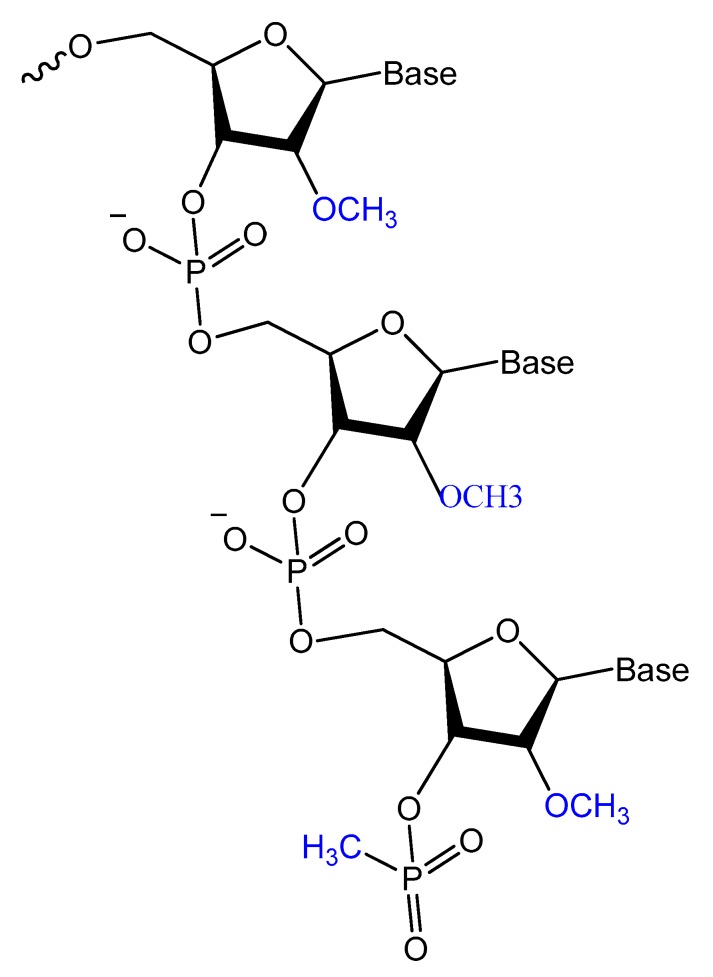
General structure of oligo-2′-*O*-methylribonucleotide containing a single methylphosphonate linkage at the 3′ end.

Prater and Miller reported a single methylphosphonateinternucleotide linkage at the 3′-end of an oligo-2′-*O*-methylribonucleotide (Figure 12) that showed high binding affinities for their complementary targets and prevented degradation by the 3′-exonuclease activity found in mammalian serum [107].

A comparison of duplex stabilities between different phosphorothioate, methylphosphonate, and 2′-OCH_3_ RNA analogs of two self-complementary DNA 14-mers was conducted by Kibler-Herzog et al. Highly modified phosphorothioates or methylphosphonates are less stable than their partially modified counterparts, which are less stable than the unmodified parent compounds. Phosphorothioate derivatives were found to be more stable when the linkage modified was between adenines rather than between thymines [108].

The effect of chemical modifications on the thermal stability of different G-quadruplex-forming oligonucleotides was investigated by Sacca et al. The methylphosphonate-modified 15mer oligonucleotide, known as the thrombin-binding aptamer (15TBA-M) gave no observable thermal transition, resulting in a flat thermal profile. In contrast, the unmodified oligonucleotide, phosphorothioate aptamers (15TBA-S), and the 2′-O-methyl ribonucleotide analogs (15TBA-O) gave a reversible and concentration-dependent thermal transition. In addition, loss of the negative charge at the level of the phosphate backbone, as in the methylphosphonate analogs, leads to a strong destabilization of the G-quadruplex structure [14].

#### 3.3.2. Triazole Modification

Oligonucleotide triazole modification instead of phosphodiester linkage has been investigated extensively in many studies [109,110] to protect the oligonucleotides from nuclease hydrolysis [111].

**Figure 13:**
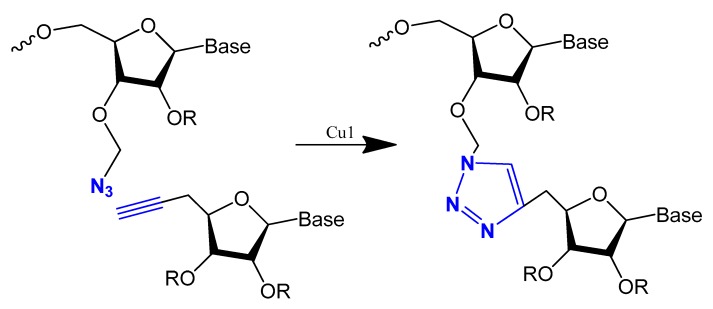
The copper-catalysed alkyne–azide cycloaddition reaction between an azide and a terminal alkyne to produce a 1,4-triazole.

**Figure 14:**
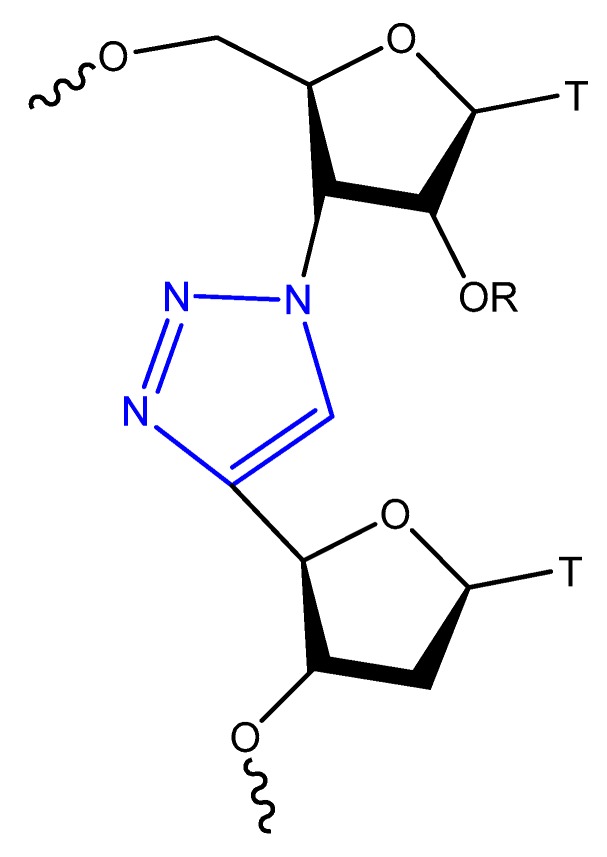
Triazol-modified thymidine dinucleotides.

This modification is usually achieved using a click reaction between azide- and alkyne-bearing nucleosides (Figure 13) [112,113] or through automated phosphoramidite synthesis with modified dinucleoside blocks (Figure 14) [114].

The triazole unit can link nucleotides directly or with methylene or single ether bond linkage. These analogs are similar to oligonucleotides and show increased resistance to nuclease cleavage. The triazole oligonucleotide analogs demonstrated DNA binding affinities similar to those of unmodified oligonucleotides. The modification was shown to protect oligonucleotides from nuclease hydrolysis [111].

Triazole-modified DNA aptamers with a structure similar to thrombin-inhibiting G-quadruplexes, TBA15 and TBA31, had been tested for their stabilities and binding affinities. No change was observed in their binding affinities, but the triazole modification protected aptamers from nuclease hydrolysis and increased their stabilities [110,115].

### 3.4. Modifications on the Bases and SOMAmers

**Figure 15:**
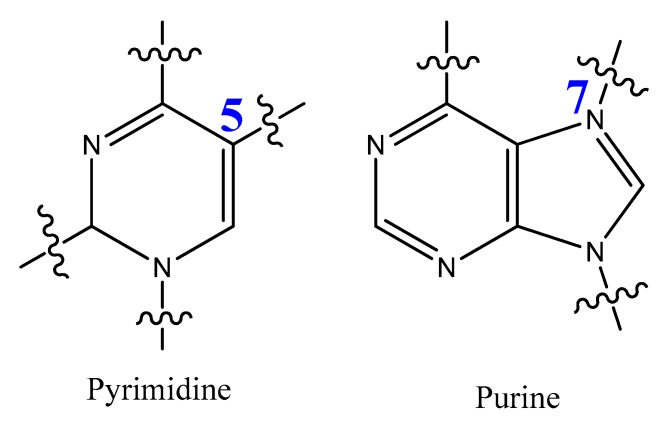
Positions of pyrimidine and purine modification.

The most modified positions on nucleic acid bases occur on pyrimidines’ C5-position and the N7 position of purines (Figure 15). These sites have been shown to be in good contact with polymerase enzymes and are easily adapted in the major groove of nucleic acid duplexes [116].

These modifications on nucleotides involve, for example, the coupling of l-proline-containing residues, dipeptide, urea derivative, and a sulfamide residue, followed by triphosphorylation. These modified 2′-deoxyribonucleoside triphosphates, dNTPs, were shown to be excellent substrates to be incorporated into DNA by the polymerase chain reaction (PCR) and are excellent candidates for SELEX [117].

**Figure 16:**
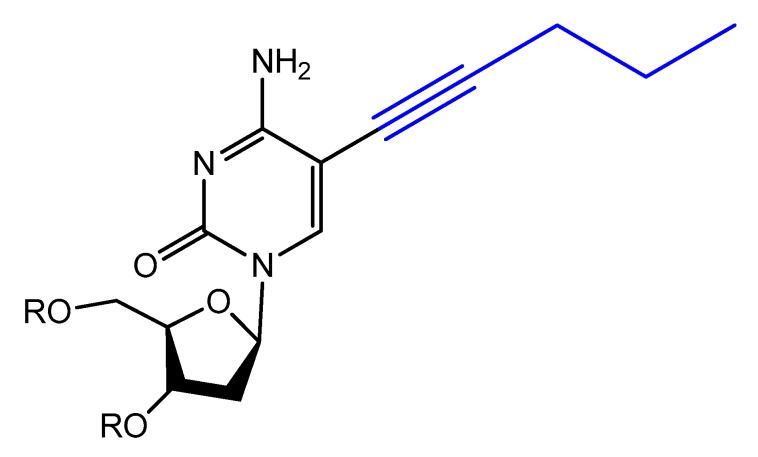
Structure of modified 5-(1-pentynyl)-2′-deoxyuridine used in aptamer selection.

Modified base aptamers are able to retain target binding properties, and thus they may enhance the binding affinity [118,119]. For example, a base-modified aptamer, 5-(1-pentynyl)-2′-deoxyuridine, used instead of thymidine, was isolated via a selection experiment against human coagulation protease thrombin (Figure 16) [118]. 

Gupta et al. introduced a different chemical modification by adding new side chains at the 5-position of uracil. These side chains ranged from high hydrophilic to more hydrophobic fragments. They assessed the impact of these side chains on the plasma pharmacokinetics of the modified aptamers.

These changes were effective in increasing the chemical diversity of the aptamers. By increasing the rate of discovery of high-affinity ligand to protein targets, they also caused an increase in nuclease resistance, with lower renal clearance for more hydrophilic side chains [119].

**Figure 17:**
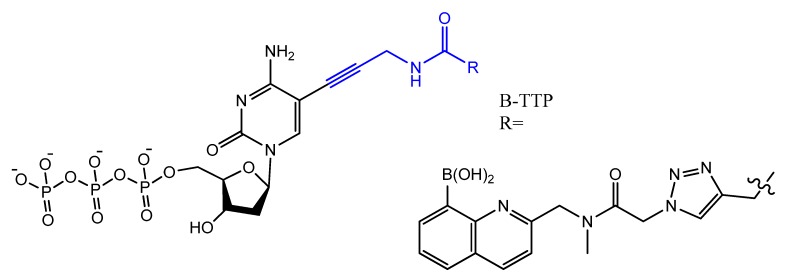
The chemical structures of B-TTP.

Photo-reactive chromophore 5-iodo-UTP was incorporated in a SELEX to generate a base-modified aptamer with a high capability for covalent interaction with HIV-1 Rev protein [120]. An anti-fibrinogen base aptamer modified with boronic thymidine-5′-triphosphate (Figure 17) was isolated by Li et al. This aptamer has specific recognition of fibrinogen glycosylation, enhancing the binding affinity compared to unmodified aptamer [121].

**Figure 18:**
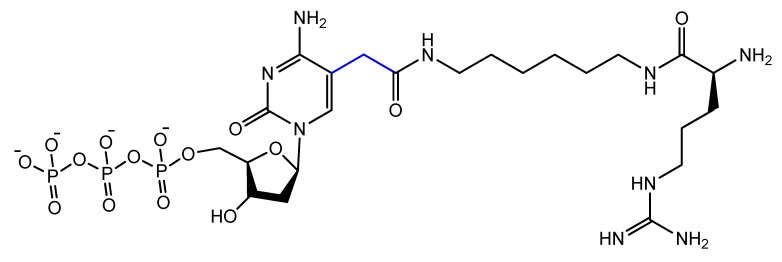
Chemical structure of the arginine-modified analog of dUTP.

The addition of an adenine residue to the C5 position of uracil ((E)-5-(2-(*N*-(2-(*N*^6^-adeninyl)ethyl)) carbamylvinyl)-uracil) increased the hydrogen bonding interaction and enhanced its efficiency to target the anticancer agent, camptothecin derivative 1 (CPT1). A very potent aptamer, CMA-70, was selected, and then improved to the shorter (CMA-59) aptamer. An improved binding affinity was seen for both modified aptamers compared to the natural aptamers [122]. Moreover, enantioselective base-modified aptamers isolated by SELEX were capable of binding only to the (*R*)-isomer of thalidomide. The aptamer thymidine was replaced with a modified deoxyuridine with a cationic group via a C5 hydrophobic methylene linker. The additional functional group improved the stability against nucleases and increased the binding affinity to thalidomide [123]. An arginine-modified dUTP (Figure 18) was involved in a SELEX experiment to improve its enantioselectivity. The isolated aptamers displayed enantioselective binding to the negatively charged glutamic acid as the target [124].

A glycol-DNA aptamer was produced from an alkyne unit, 5-ethynyl-modified dUTP, via SELMA selection could recognize the monoclonal antibody, 2G12, which is known to bind to mannose-rich glycans on the HIV envelope protein, gp120, thus neutralizing various HIV strains [125,126,127].

Lee and colleagues revealed that 5-BzdU (5-(*N*-benzylcarboxyamide)-2-deoxyuridine) modification of the AS1411 aptamer might selectively increase its targeting affinity to cancer cells while having no significant influence on the normal healthy cells [128]. The 5-BzdU residue was further modified by replacing the benzyl group by other aromatic or aliphatic groups to enhance the binding affinity of this modified aptamer to their targets [129].

**Figure 19:**
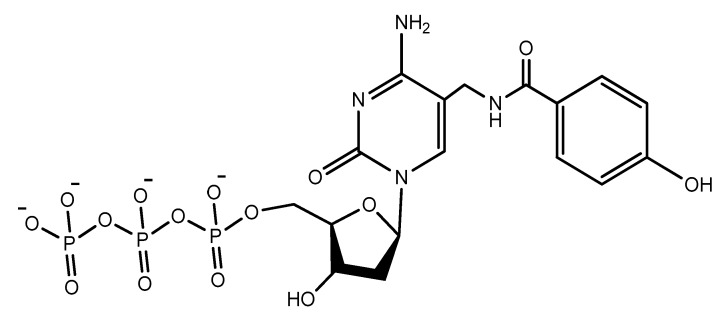
The modified phenol-dUTP nucleotide.

Another increasingly expanding approach utilizes the replacement of natural nucleotides with artificial unnatural bases in the DNA sequence to improve the therapeutic properties [130,131]. A nucleoside triphosphate modified with a tyrosine-like phenol (Figure 19) was used in the selection of DNA aptamers against *Escherichia coli* DH5α cells. The modified aptamer displayed high selectivity and affinity for the target cells compared to the unmodified aptamer [132].

**Figure 20:**
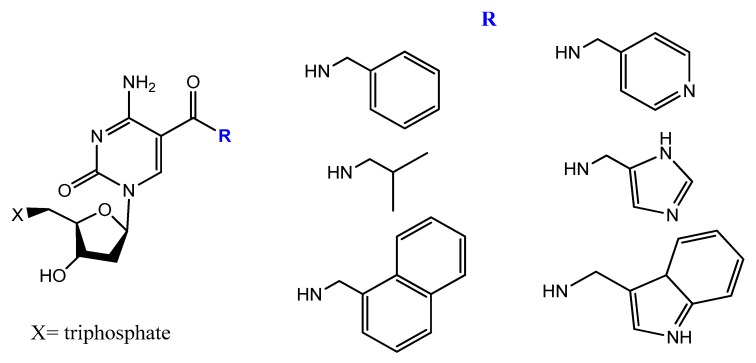
New dUTP derivatives prepared by Vaught et al. [134].

5-[(*p*-Carborane-2-yl)ethynyl]-2′-deoxyuridine 5′-*O*-triphosphate was synthesized and used by Balintová et al. as substrate for KOD XL DNA polymerase in a primer extension (PEX) reaction to generate carborane-modified DNA or oligonucleotides. These carborane-modified hydrophobic aptamers may increase the potential interactions against hydrophobic proteins or analytes [133]. C5-modified carboxamide pyrimidines’ functionality was a smart choice to facilitate the attachment of other hydrophobic groups, such as benzene, thiophene, naphthalene, isopropyl, and amino acid derivatives (Figure 20) [134].

A new protocol was lately described to select nucleobase-modified aptamers. This protocol utilizes click chemistry (CuAAC) to introduce the favored nucleobase modification based on alkyne-modified uridine (5-ethynyl-deoxyuridine (EdU)) instead of thymidine. This new protocol enables a wide range of functionality and generates modified DNA aptamers with extended interaction properties [135].

The slow off-rate-modified aptamers (SOMAmers) are aptamers with significant base modification to give a protein-like functionality. This formulation improves the binding affinities and binding kinetics with enhanced selectivity when compared to traditional aptamers. This is achieved by increasing both the number and strength of the hydrophobic interactions between nucleic acids and the corresponding targets, thus partially mimicking the binding mode of antibodies and other proteins. The power of this kind of base modification is that it exhibits very little nuclease degradation over a 48-h incubation in human serum [136,137], it facilitates the detection of various proteins in the blood serum, and it has been widely applied in the discovery of disease biomarkers [138,139].

Modified DNA SOMAmers ((5-(*N*-benzylcarboxamide)-2′-deoxyuridine (Bn-dU) or 5-[*N*-(1-naphthylmethyl)carboxamide]-2′-deoxyuridine (NapdU) replacing dT) that inhibit interleukin-6 (IL-6) signaling, a key component of inflammatory diseases, were found to be stable in serum and blocked the interaction of IL-6 with its receptor, IL-6Rα [136].

An advanced SOMAmer-based assay was developed for quantification of soluble glypican-3 in hepatocellular carcinoma (HCC) patient samples using glypican-3 SOMAmer. The assay verified its good sensitivity, accuracy, and precision compared to the traditional antibody-based assay, with a high binding affinity [140]. Gawande and co-workers explored selection experiments using double-modified DNA aptamers with amino-acid-like moieties on pyrimidine bases to target proprotein convertase subtilisin/kexin type 9. They isolated aptamers that showed higher affinity, biostability, and inhibitory potency compared to singly modified aptamers with broad utility in research, diagnostic, and therapeutic applications [141].

Wang et al. reported a biophysical and enzymatic properties study of three widely used protein-like side chain dNTPs: 8-histaminyl-deoxyadenosine (dAimTP), 5-guanidinoallyl-deoxyuridine (dUgaTP), and 5-aminoallyl-deoxycytidine (dCaaTP). The base-pairing abilities of oligonucleotides having one or three modified nucleosides were tested by thermal denaturation analysis and as a substrate for enzymatic polymerization with both modified and natural dNTPs [142].

### 3.5. Spiegelmers

**Figure 21:**
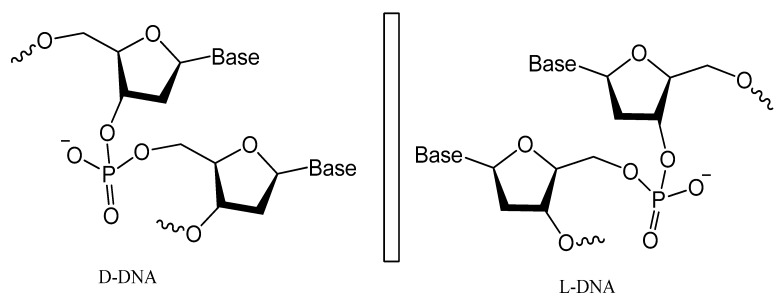
Structures of l-deoxyoligonucleotide (l-DNA). Mirror image aptamers are composed of non-natural l-ribose nucleotides.

Spiegelmers are the synthetic mirror image of d-nucleic acids that show high resistance to nuclease degradation and may retain their binding affinity to their d-form targets or be selected with high binding affinity to new targets (Figure 21) [143]. For example, NOX-A12, a structured mirror image RNA oligonucleotide in the l-configuration that neutralizes stromal cell-derived factor-1, interferes with chronic lymphocytic leukemia migration and drug resistance [144]. NOX-A12, a spiegelmer that binds and neutralizes CXCL12, was developed for interference with CXCL12 in the tumor microenvironment and for cell mobilization.

An l-RNA aptamer targeting the HIV-1 trans-activation responsive (TAR) RNA was developed. This spiegelmer showed great specificity and strong binding activity based on tertiary interactions more than Watson–Crick pairing [145]. In addition, NOX-G15 is a mixed DNA/RNA mirror image aptamer that binds to the glucagon and improves glucose tolerance in models of type 1 and type 2 diabetes [146].

A 67-mer l-enantiomeric spiegelmer for gonadotropin-releasing hormone (GnRH) was selected from a random pool of oligonucleotides, and this effective antagonist spiegelmer showed a high binding affinity (K_D_ = 20 nM) with longer plasma half-life stability [147]. Another l-GnRH spiegelmer was chemically synthesized according to the isolated natural d-GnRH aptamer. The resulting spiegelmer had similar affinities to that of d-aptamers [148]. A biologically stable mirror image enantiomeric l-DNA spiegelmer against bacterial Staphylococcal enterotoxin B was developed. The spiegelmer bound the whole protein target, with only a slightly reduced affinity, which shows the possibility of identifying spiegelmers against large protein targets [149]. Spiegelmers also undergo similar different strategies and chemical modifications as natural aptamers to enhance their stability against nucleases and improve their binding affinity [143]. A nuclease-resistant modified l-RNA aptamer (MLRA) with cationic nucleotide, 5′ aminoallyl-uridine, was isolated in an in vitro selection process and this spiegelmer was capable of binding oncogenic pre-miR-19a with exceptional affinity, and the cationic modification was absolutely crucial for binding [150].

Finally, Taylor and Holliger described protocols for the replication of artificial analogs of DNA and RNA having a different backbone or sugar homologous xeno nucleic acids (XNAs). For the directed evolution of synthetic oligonucleotide ligands (XNA aptamers) for specific targeting of proteins or nucleic acid units, a cross-chemistry selective exponential enrichment (X-SELEX) approach is used. This approach may be applied to select and isolate fully modified XNA aptamers for a wide range of target molecules [151].

Conventional SELEX, based on only four natural DNA/RNA nucleotides, often yields poor binders only. Synthetic biology has increased the number of DNA/RNA building blocks, with tools to sequence, PCR amplifies, and clone artificially expanded genetic information systems (AEGISs). Several examples have been reported of a SELEX using AEGIS, producing a molecule that binds to cancer cells [130,152].

A functional RNA molecule containing an artificial nucleobase pair was designed by Hernandez et al. to increase the number of building blocks in nucleic acids.They replaced the C:G pair by a pair between two components of an artificially expanded genetic-information system (AEGIS), Z and P (6-amino-5-nitro-2(1*H*)-pyridone and 2-amino-imidazo [1,2-a]-1,3,5-triazin-4-(8*H*)-one). The structure shows that the Z:P pair does not greatly change the conformation of the RNA molecule nor the details of its interaction with a hypoxanthine ligand, with a 3.7 -nM affinity of the riboswitch for guanine [153].

A laboratory in vitro evolution (LIVE) experiment based on an artificially expanded genetic information system (AEGIS) was reported by Biondi et al. An AEGIS aptamer that binds to an isolated protein target was outlined against an antigen from *Bacillus anthracis*. The AEGIS aptamer showed improved stability and binding of the aptamer to its target [154].

### 3.6. Circular Aptamers

The majority of nucleic acid degradations are caused by plasma exonucleases that break the phosphodiester bonds at either the 3′ or 5′ terminals, leading to the cleavage of of nucleotides one at a time. Chemical modifications on the terminals increase the stability; however, cyclization eliminates this source of degradation entirely [155]. For example, a comparison between two linear aptamers targeting MUC1 and HER2 with their distinct double strand circular aptamers showed significant biostability for the circular ones [20].

Circular bivalent aptamers (cb aptamers) were constructed from aptamers selected against live cancer cells, and were tested for their nuclease stability, binding affinity in vivo, and for their thermal stability [156]. The results showed that circular aptamers sustained their sequence integrity for 12 h compared to 1 h for linear aptamers in biological media. In addition, the thermal stability was enhanced by at least 10 °C. Another example is a cyclic thrombin-binding aptamer (cycTBA), which was prepared by covalently bonding the 3′ and 5′ ends of the linear aptamer via a linker [157]. The thermal stability of cycTBA was highly enhanced (an increase of the melting point by 18 °C in both the K^+^ and Na^+^ ion environment) and a 180-fold increase in the half-life in PBS, which is a strong indicator that cycTBA has a higher resistance towards nucleases. However, its anticoagulant activity dropped by half. Such a drop in the activity is an indication of a need for some flexibility in the aptamers’ structure [158].

### 3.7. Multivalent and Dimerization of Aptamers

As mentioned before, increasing aptamers′ affinity to the target and their stability are the main hurdles for aptamers′ applications, especially as therapeutics. The several chemical modifications to increase stability via increasing the size and mass of aptamers are promising. However, it might affect the affinity towards targets. Multivalent aptamers might be the solution, since it increases the size and at the same time increases the affinity. Multivalent aptamers are constructs composed of two (dimer) or more (multi) identical or different aptamer motifs, with or without additional structural elements [159]. Simply, connecting identical aptamers should increase affinity to its target since it increases the number of contact points. The connection of different aptamers can also lead to an increase in versatility [160].

Further refinement of aptamers is needed to achieve desired affinities [161]. Dimerization of aptamers (identical and different) was performed by Hasegawa and co-workers [162]. Dimers of two aptamers against thrombin (each binds to a different site) using a thymine linker with variable length proved an enhanced affinity compared to the monomers in addition to an improved thrombin-inhibiting effect. They also tested a dimer of two identical aptamers against vascular epithelial growth factor (VEGF_165_), which is a dimeric protein. Ligand-guided selection (LIGS) of aptamers is known to give aptamers with high specificity; however, these aptamers suffer from low affinity, which hinders their further application in diagnostics and therapeutics. For example, an LIGS-aptamer against membrane IgM (mIgM) was introduced with high specificity. In order to improve its affinity, a dimeric aptamer was prepared that showed enhanced affinity without affecting its specificity [163].

Multivalency not only enhances the affinity and stability of aptamers but it can also improve cellular uptake. Multivalent DNA structures with dual aptamers, a guanosine-rich oligonucleotide 100 aptamer (AS1411), which was developed to target nucleolin-overexpressing cells, and mucin-1 (MUC-1) aptamers, which were developed to target mucin glycoproteins, showed superior intercellular uptake compared to oligomers with a single type of aptamers [21].

Extensive efforts have been dedicated to developing fluorescent RNA aptamers, which are crucial to facilitate live-cell imaging. Fluorescent RNAs were developed. However, these aptamers suffered from poor brightness and photostability. A dimerized aptamer (*o*-Coral) was prepared and tested, showing high affinity, brightness, and stability compared to its parent aptamer [22].

## 4. Chemical and Physical Conjugation Strategies of Aptamers to Nanoparticles

Nanoparticles can be functionalized with different types of aptamers as targeting ligands. This functionalization can be achieved via various approaches without affecting the 3-d aptamer functionality. Chemical covalent bonds and physical conjugation strategies were frequently used through a spacer or a linker to maintain aptamer binding activity.

### 4.1. Direct and Post-Insertion

Direct aptamer conjugation in the nanoparticle formulation is usually done by certain modifications on the aptamer structure. For example, an aptamer called TDO5, which was selected specifically to Ramos cells (a B-cell lymphoma cell line), was incorporated in micelle construction by attaching a simple lipid tail phosphoramidite with diacyl chains onto the end of the aptamer inserted with a PEG linker. This amphiphilic unit is self-assembled into a spherical micelle structure. The results showed an 80-fold higher internalization of the TD05-micelles by Ramose cells compared to unconjugated micelles [164].

**Figure 22:**
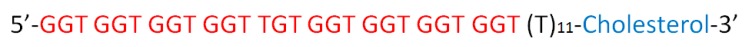
Schematic illustration of the NCL aptamer conjugated at 3′ with cholesterol.

Direct conjugation was also used with anti-nucleolin-specific DNA (NCL) aptamer (AS1411) in Xing et al.’s study to functionalize the surface of doxorubicin-loaded liposomes. The AS1411 aptamer was firstly bound to a cholesterol molecule through a poly-thymine spacer and then involved in the liposome bilayer structure during liposome preparation (Figure 22) [165]. Similarly, AS1411 aptamer direct conjugation was used to coat liposomes loaded with cisplatin [166].

Riccardi et al. designed a highly integrated and multifunctional nanosystem based on niosomal formulations. These niosomes were loaded with the nucleolipid Ru(III)-complex, HoThyRu, and then decorated with AS1411 aptamer via the post-insertion dispersion method. These niosome formulations showed increased antiproliferative activity when loaded with both the Ru(III)-complex and the AS1411 aptamer compared with all the tested controls. A valuable therapeutic window was found for the HeLa cancer cells for concentrations up to 3.5 μM. The final formulation provides several medical applications, including “on-demand” release, specific tissue/cell type targeting, in vivo imaging, and diagnosis [167].

Aptamers can also be directly incorporated in the nanoparticle formulation. For example, gadolinium-doped luminescent and mesoporous strontium hydroxyapatite nanorods loaded with doxorubicin (Gd:SrHap-Dox) were coated with AS1411 aptamer. The coating was performed by fridge incubation of preformed drug-loaded nanorods with a G quadruplex structure of the AS1411 aptamer for 12 h, mainly through strong electrostatic forces. Aptamer-capped Gd:SrHapnanorods can be internalized into MCF-7 cells, resulting in pore opening and drug release [168].

**Figure 23:**
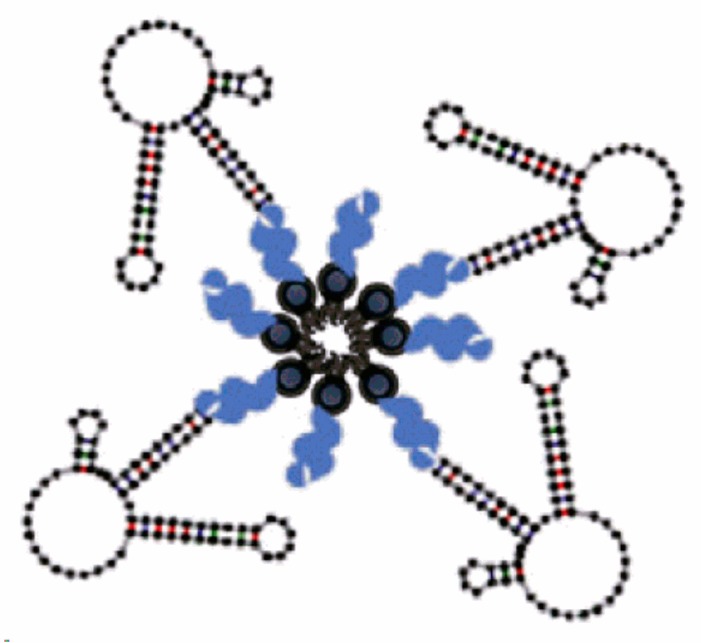
DSPE-PEG-Apt1 micelles [49].

Post-insertion is mostly used to functionalize preformed liposomes with certain aptamers linked to a the lipid anchor, such as DSPE-PEG, to form micelles (Figure 23) [49].

Willis et al. functionalized the membrane of lipid vesicles with nuclease-stable, anti-VEGF aptamer, using the diacylglycerol (DAG) lipid as an anchor. This conjugation did not influence the binding affinity of the aptamer, and the plasma residence time of the liposome-anchored aptamer was considerably improved compared with that of the free aptamer [64].

### 4.2. Carbodiimide Chemistry

**Figure 24:**
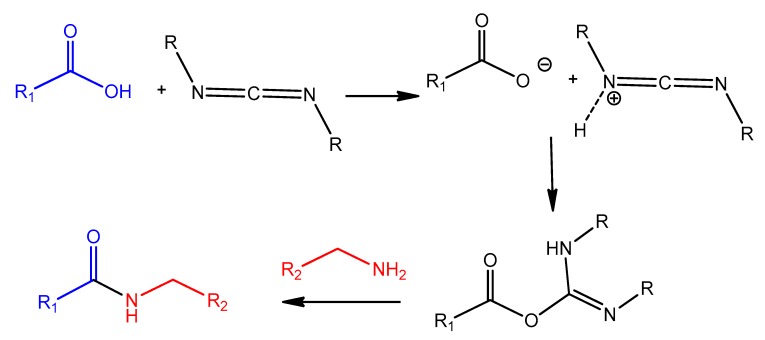
The formation of a covalent amide bond via carbodiimide coupling.

Carbodiimides are frequently used in organic synthesis, bioconjugation, and drug delivery. For example, the water-soluble carbodiimide, 1-ethyl-3-(3-(dimethylaminopropyl)-carbodiimide (EDC), is a common reagent used for activating carboxylic acid residues to react with ligands containing amino groups, resulting in amide bond linkage (Figure 24) [169].

Mann et al. functionalized liposomes with oligonucleotide aptamer (thioaptamer) against E-selectin (ESTA) using carbodiimide chemistry. The carboxylated Cy3-labeled or unlabeled ESTA was conjugated to amino PEGylated stealth liposome (NH_2_-PEG-lip) using 1-(3-dimethylaminopropyl)-3-ethylcarbodimide hydrochloride (EDC) and sulfo(*N*-hydroxysuccinimide) (sulfo-NHS). In Vitro targeting studies verified efficient and rapid uptake of the ESTA-conjugated liposomes (ESTA-lip). Moreover, the aptamer-liposomes were retained in a human breast tumor xenografted model without any decrease in the circulation half-life [170].

AS1411 aptamer-modified thermosensitive liposome (TSL) was designed as an efficient magnetic resonance imaging (MRI) probe. Zhang et al. encapsulated Gd-chelates into an optimized TSL formulation, followed by conjugating with AS1411 for specific targeting against tumor cells that overexpress nucleolin receptors. The TSLs’ structure included carboxylate DSPE-PEG2000 (DSPE-PEG2000-COOH), where the AS1411 aptamer was conjugated onto TSL through EDC/NHS carbodiimide chemistry. The resulting liposomes exhibited much higher T_1_ relaxivity in MCF-7 cells, which enhances early cancer diagnosis [171].

A pH-sensitive polymeric micelle using d-α-tocopheryl polyethylene glycol-block-poly-(β-amino ester) (TPGS-b-PBAE, TP) as a pH-sensitive copolymer, loaded with paclitaxel (PTX), and functionalized by AS1411 on the surface, was developed by Zhang et al. [172]. The aptamer conjugation was achieved by carbodiimide chemistry, where the TPGS polymer was treated with succinic anhydride to obtain carboxyl-modified TPGS polymer, then an amine-modified AS1411 aptamer was added to the EDC/NHS-activated TPGS polymer. A higher cellular uptake, significant cytotoxicity, reduction in tumor growth, and myelosuppression were observed in in vitro and in vivo in mice SKOV3 ovarian cancer cells compared with free PTX injection [172]. As another similar example, a polymeric micelles-AS1411 aptamer functionalization based on the same carbodiimide chemistry was used in designing a multifunctional composite micelle made of poloxamer (Pluronic^®^ F127) and beta-cyclodextrin-linked poly(lactide)-copoly(ethylene glycol) (PLA-PEG) encapsulating doxorubicin [173]. Aptamer AS1411-modified Pluronic F127 (Pluronic F127-Ap) was synthesized by the reaction of carboxylated-Pluronic F127, with the amino groups at the ends of AS1411 aptamers. An in vivo study in MCF-7 tumor-bearing mice demonstrated that the AS1411-functionalized composite micelles showed increased blood circulation, enhanced accumulation in tumor, improved anticancer activity, and decreased cardiotoxicity [173]. Moreover, the same carbodiimide protocol for aptamer conjugation was adapted in developing a doxorubicin-loaded unimolecular micelle composed of hyperbranched copolymer molecules of H40 and PLA-PEG polymers with an anti-PSMA aptamer covering the surface. This formulation showed a higher level of DOX accumulation in the tumor tissue compared to aptamer-free polymeric micelles [174].

Polymeric nanocarriers, composed mainly of synthetic polymers, such as polyesters or cationic polymers, as well as of natural polymers, such as polysaccharides or proteins (albumin, collagen) [175], can also be functionalized by aptamers through carbodiimide chemistry. This is achieved by carboxyl functionalization of the hydrophilic part of the polymeric unit (PEG) with carboxyl groups on the nanoparticle surface, making them available for surface carbodiimide coupling with 5′-amino-aptamer [176]. For example, carboxy-terminated poly(d,l-lactic-co-glycolic acid)-block-poly(ethylene glycol) (PLGA–b–PEG–COOH) polymer nanoparticles were conjugated to the A10 RNA aptamer (Apt) that binds to prostate-specific membrane antigen (PSMA), and this formulation was evaluated in a LNCaP (PSMA+) xenograft mouse model of prostate cancer [176]. Non-aggregated polymeric nanoconjugates of paclitaxel–polylactide (Ptxl–PLA) were prepared. The PLA was functionalized with PEG-COOH to obtain PLA-PEG-COOH, which was bioconjugated with the amine-terminated A10 aptamer through the carbodiimide coupling reaction in the presence of EDC and NHS to give aptamer-PLA-PEG-COOH/Cy5-PLA nanoconjugates. This aptamer–nanoconjugate was found to be able to effectively target prostate-specific membrane antigen in a cell-specific manner [177]. The same carbodiimide coupling chemistry of PLA-PEG-COOH copolymer with amino terminal-aptamer has also been applied in decorating docetaxel (Dtxl)-encapsulated nanoparticles. It was formulated with biocompatible and biodegradable PLGA-b-PEG copolymer, with the A10 2′-fluoropyrimidine RNA aptamers to distinguish the extracellular domain of the prostate-specific membrane antigen (PSMA) [178].

**Figure 25:**
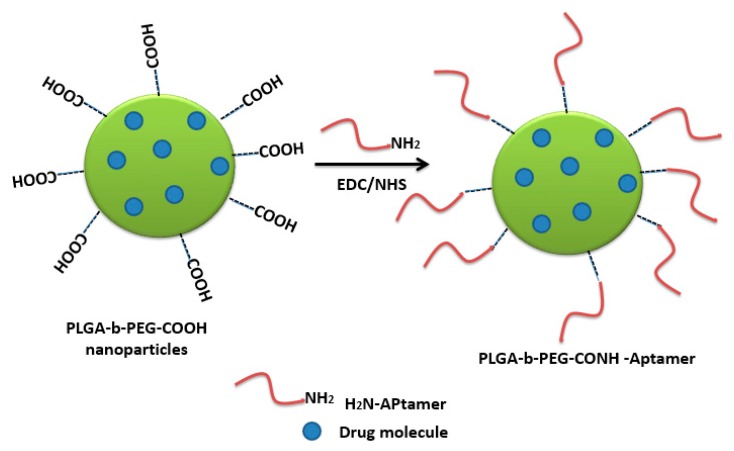
Carbodiimide bioconjugation approaches of aptamer functionalized on the surface of PLGA-b-PEG-COOH-based nanoparticles.

Carbodiimide bioconjugation approaches of aptamer functionalized on the surface of PLGA-b-PEG-COOH-based nanoparticles have been developed for targeted drug delivery systems, such as anti-NCL aptamer (AS1411) [179], anti-MUC1 aptamer [180], and epithelial cell adhesion molecule (EpCAM) [181] for paclitaxel-targeted delivery to glioma, paclitaxel-targeted delivery to breast cancer, and for curcumin- and Nutlin-3a-targeted delivery to colorectal adenocarcinoma, respectively (Figure 25) [49].

**Figure 26:**
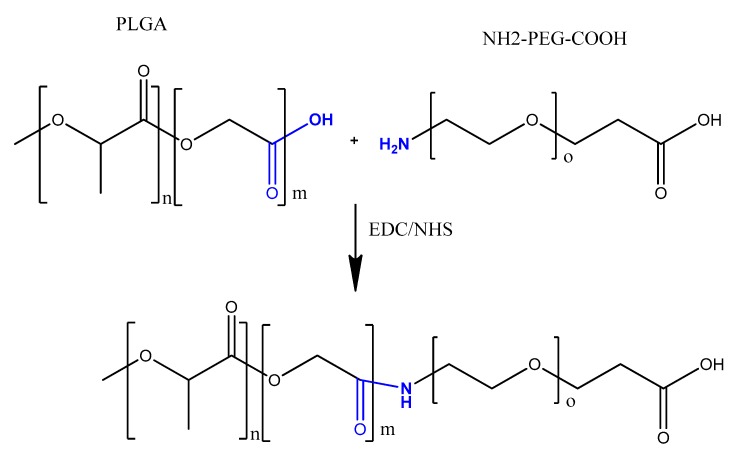
PLGA-b-PEG copolymer synthesis by EDC/NHS carbodiimide coupling chemistry [182].

Alibolandi et al. adapted the same approach to synthesize PLGA-b-PEG nanoparticles loaded with doxorubicin (Figure 26), followed by anti-EpCAM aptamer functionalization by similar coupling chemistry, showing a higher tumor inhibition in a mouse xenograft model of human small lung cancer compared to non-aptamer-conjugated polymeric nanoparticles [182].

PLGA-b-PEG nanoparticles encapsulating salinomycin were also decorated with a targeting RNA aptamer that binds the CD133 marker, based on carbodiimide coupling chemistry. Higher cytotoxicity towards CD133+ osteosarcoma cancer stem cells was shown compared to the non-functionalized nanoparticles [183]. AS1411 aptamer-tagged PLGA-lecithin-PEG nanoparticles loaded with paclitaxel for tumor cell targeting and delivery were tested by Aravind et al. The functionalized PLGA-lecithin-PEG nanoparticles exhibited high encapsulation efficiency and superior sustained drug release compared to the drug loaded in plain PLGA nanoparticles. They are considered a potential carrier candidate for differential targeted drug delivery [184]. To treat MUC1-overexpressing adenocarcinomas, chitosan-based polymeric nanocarriers for 5-fluorouracil [185] and SN38, an irinotecan metabolite [186], targeted with a MUC1 aptamer via carbodiimide chemistry (EDC/NHS technique), were performed. These aptamer-guided nanocarriers showed enhanced cytotoxicity compared to non-targeted nanocarriers [185,186].

AS1411 aptamer-Ag nanoclusters were conjugated to polyethylene glycol-coated ultrasmall gadolinium oxide nanoparticles (PEG-Gd_2_O_3_ NPs) through a covalent linkage between the carboxyl group (−COOH) of PEG and the amino group (−NH2) modified on the 5-end of AS1411 aptamer. These tracking-imaging nanoparticles induced hyperthermia by targeting MCF7 cancer cell lines [187].

**Figure 27:**
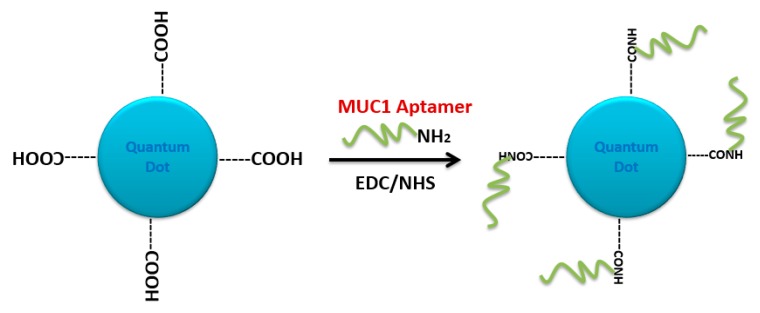
Synthesis of quantum dot-MUC1 aptamer [28].

Carbodiimide coupling chemistry was also applied for conjugating aptamers to quantum dot (QD) nanoparticles. Carboxyl core-shell CdSe/ZnS QD was first activated with EDC/NHS reagents. The resulting N-hydroxysuccinimide-activated QD was covalently linked to 5′-NH_2_-modified A10 PSMA aptamer. Doxorubicin then intercalated in the double-stranded stem of the A10 aptamer (QD-Apt(Dox)). This multifunctional nanosystem can deliver Dox to targeted prostate cancer cells and sense the delivery of Dox by activating the fluorescence of QD [188]. A similar conjugation chemistry was adapted by designing a tumor-targeted pH-responsive quantum dot-mucin1 aptamer-doxorubicin (QD-MUC1-DOX) conjugate for the chemotherapy of ovarian cancer. It was shown that the developed aptamer-guided conjugate had higher cytotoxicity than free DOX in multidrug-resistant cancer cells (Figure 27) [28].

The same 5′-amine-modified MUC-1 aptamer was used to cross-link carboxyl-functionalized silica nanoparticles (COOH-FSiNPs) by EDC/NHS carbodiimide chemistry. This aptamer-conjugated Rubpy-doped silica nanoparticle was tested for human breast carcinoma MCF-7 cells’ labeling. The dye-doped silica nanoparticles serve as a stable bioprobe because of their facile conjugation with the desired biomolecules [189].

5′-NH2-modified A10 PSMA aptamer was also conjugated to the surface of carboxyl-modified superparamagnetic iron oxide nanoparticles via carbodiimide chemistry. Doxorubicin intercalates within the aptamer GC pair. This nanocomposite combination showed higher toxicity to targeted cells and minimized side effects to non-targeted cells [190].

3′-NH_2_-modified AS1411 aptamer was covalently linked to an EDC/NHS-activated nanocluster composed of a mesoporous metal-organic framework (MOF) shell, and an upconversion luminescent core (UCNP). This targeted nanocluster was further intercalated with doxorubicin to form a UCNPs/MOF-Dox-AS1411 multifunctional nanosystem that intervenes drug delivery and cell imaging [191].

A new material that incorporates gold nanorods with a mesoporous silica structure that has been surface modified with DNA aptamer was constructed. This nanostructure was functionalized with a carboxylic group using succinic anhydride. Subsequently, the aptamer-gated nanovehicles were decorated through binding with amine-modified AS1411 aptamer using EDC/NHS carbodiimide chemistry. This multifunctional nanostructure combined chemotherapy, photochemotherapy, and imaging into one system [192].

Standard peptide bond formation methodology using EDC/NHS coupling chemistry was also used to conjugate amino-modified anti-protein tyrosine kinase 7 aptamer (anti-PTK7 aptamer) (sgc8) aptamers on the surface of carboxyl-free-modified porous hollow magnetite nanoparticles (PHMNPs) loaded with doxorubicin. This multifunctional nanosystem was tested for targeted cancer chemotherapy and magnetic resonance imaging (MRI) [193].

### 4.3. Thiol Maleiimide and Related Chemistry

**Figure 28:**
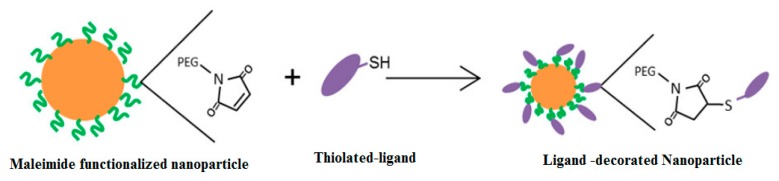
Thiol maleimide coupling chemistry [27].

Thiol maleimide coupling chemistry or Michael addition of a thiol to a maleimide or any Michael accepter is commonly used for bioconjugation of thiolated (-SH) drugs or targeting ligand to macromolecules or on the surface of nanoparticle drug delivery systems (Figure 28) [27].

A DNA aptamer (sgc8) was successfully attached to the surface of liposomes using the thiol–maleimide chemistry [194]. To prepare the sgc8 aptamer-liposomes, a maleimide polyethyleneglycol (PEG-Mal) was incorporated in the liposome membranes during the liposomes′ preparation, followed by overnight incubation at 4 °C with 5′-thiolated-sgc8-TMR aptamer. The sgc8 aptamer-liposome cellular uptake studies demonstrated that the targeting was critical for cellular uptake [194].

**Figure 29:**
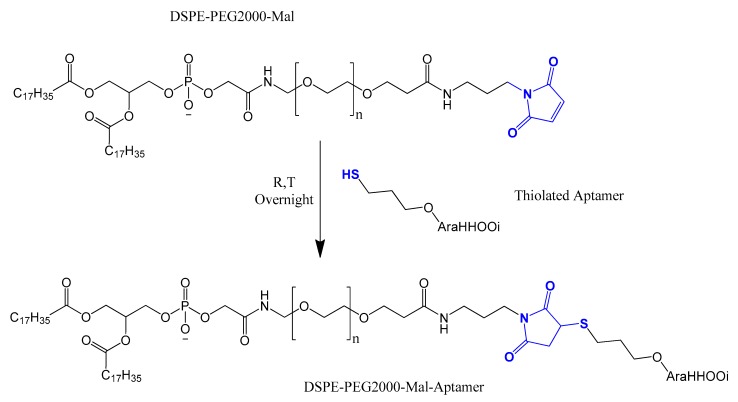
The reaction of thiol-modified aptamer AraHH001 with maleimide-PEG2000-DSPE.

A DNA aptamer was selected against mouse tumor endothelial cells (anti-mTEC aptamer) (AraHH001). This aptamer was functionalized to the surface of PEGylated liposomes using the thiol–maleimide crosslinking. This functionalization indicates the potential of the targeted delivery of anti-angiogenesis drugs into tumor endothelial cells (Figure 29) [195].

Li et al. developed nucleolin-targeting liposomes guided by aptamer AS1411 to deliver anti-BRAF siRNA (siBraf) for the treatment of malignant melanomas. The AS1411 aptamer was covalently attached to siRNA-loaded lipoplexes via thiol–maleimide chemistry. This combination showed major silencing activity in A375 tumor xenograft mice and inhibited melanoma growth [196].

An RNA aptamer (Apt1) against the CD44 receptor was selected [197] and conjugated to the surface of PEGylated liposomes using the thiol–maleimide chemistry. These Apt1-functionalized liposomes showed higher selectivity and uptake by CD44+ cancer cell lines compared to the CD44− cell line [198]. Moreover, thiolated Apt1 conjugation with DSPE-PEG-maleimide using thiol–maleimide chemistry followed by successful post-insertion into liposomes was done by Alshaer et al. [199]. Micelles composed of DSPE-PEG and DSPE-PEG-maleimide were prepared by the thin film evaporation hydration method. Conjugation of Apt1-SH to micelle-DSPE-PEG-mal was performed using a thiol–maleimide cross-linking reaction to form a thioether bond. Then, the micelle-DSPE-PEG-Apt1 was post-inserted in siRNA-loaded liposomes by mixing and incubating at 60 °C for 1 h. Such nanocarriers showed a higher luc2 inhibition by Apt1-functionalized liposomes in vitro and a prolonged gene inhibition in vivo on an orthotopic MDA-MB-231 breast cancer model [199]. The same thiol–maleimide post-insertion protocol was also adapted with AS1411-functionalized thermosensitive liposomes encapsulating both doxorubicin and ammonium bicarbonate, for the selective targeting of multidrug-resistant breast cancer cells (MCF-7/MDR) that overexpress nucleolin [200].

Dendrimers can also be guided by aptamers. Dendrimers are synthetic, highly branched (treelike) macromolecules with nanometric dimensions. Dendrimers have many functional groups on their surface, offering a high number of sites for the conjugation of targeting ligands [201]. A gene delivery system composed of polyamidoamine (PAMAM) dendrimers was functionalized at the surface with PEG-Mal via a specific reaction between the primary amino groups of PAMAM and the NHS groups of the bifunctional PEG derivative. The resulting conjugate, PAMAM-PEG-Mal, was bioconjugated with 3′-SH-modified second generation anti-PSMA aptamer (A10-3.2) via the thiol–maleimide chemistry. This targeted nanoformulation, loaded with the tumor suppressor non-coding genes (miR-15a and miR-16-1), induces apoptosis and selective cell death of prostate cancer cells [202].

Parallel chemistry to thiol–maleimide conjugation has been used by introducing a soft electrophile (as the Michael system) at the surface of the nanoparticle. This approach is most useful in dendrimer nanoparticles due to their versatile surface functional groups [201]. Anti-nucleolin aptamer AS1411 was conjugated to an amphiphilic multimolecular hyperbranched dendritic polymer using this technique. This targeted system has enhanced cell uptake, excellent fluorescence properties, and smart targeting capability in vitro, indicating the great potential of promising carriers for bioimaging and cancer-specific delivery [201].

Another thiol–maleimide-related aptamer coupling was used in the conjugation of an EpCAM aptamer to carboxymethyl cellulose (CMC)-magnetic iron oxide nanoparticles (CMC-MNPs). CMC-MNPs interacted with 1,6-diaminohexane via carbodiimide coupling in order to introduce some amino groups to the CMC-MNPs’ surface. Next, thiolated EpCAM aptamer was covalently linked to amino-functionalized CMC-MNPs using the hetero-bifunctional crosslinker, 4-maleimidebutyric acid-NHS ester. This magnetic nanoparticle-aptamer probe was utilized for specific hepatocellular carcinoma imaging and treatment [203].

3-(2-Pyridyldithio) propionyl hydrazide (PDPH) was an alternative aptamer bioconjugation cross-linker. PDPH is a heterofunctional crosslinker that possesses a carbonyl-reactive hydrazide group at one end and a sulfhydryl-reactive-pyridyl disulfide group on the other end [204].

In a study by Pala et al., dextran-coated ferric oxide magnetic nanocarriers conjugated with the HER2 aptamers were developed to induce hyperthermia in SK-BR-3 and U-87 MG cells. The hydrazide group firstly reacted with the dextrin moiety to form a hydrazone bond, followed by conjugation with 5′-thiolate HER2 aptamer to the pyridyl end to form a disulfide bond. The aptamer-tagged nanoparticles were highly specific towards the HER2-expressing cells and a 90-fold lower dose was required to kill 50% of the targeted cells compared to the aptamer-free nanoparticle [205]. HER2 aptamer (HB5) was also attached to silica-carbon nanoparticles and loaded with doxorubicin for chemo-photothermal therapy of an HER2+ breast cancer cell line (SK-BR-3). The preparation of aptamer-functionalized mesoporous silica-carbon-based doxorubicin (MSCN-PEG-HB5) was carried out by bringing in NHS-PEG3500-MAL. PEG served as a linker between amine-functionalized MSCN and thiol group-modified HB5. The results verified higher cytotoxicity of the combined therapy compared to chemo- or photo-therapy alone [206].

### 4.4. Electrostatic and cDNA Strand Conjugation

This approach controls the number of aptamers on the surface of nanoparticles and offers more versatility to the functionalization method. This technique is based on a DNA strand conjugated on the surface of the nanoparticle that is a complementary fit to the intended aptamer [207]. Baek et al. used a complementary DNA strand (cDNA) conjugated to DSPE-PEG-maleimide phospholipids through a thiolated linker and then post-inserted it into preformed PEGylated liposomes. The anti-prostate-specific membrane antigen aptamer (anti-PSMA aptamer) was then paired to the cDNA strand conjugated on the surface of the preformed liposomes. This formulation was tested in vitro on a PSMA+ cell line (LNCaP) and on a mouse model of xenografted human prostate cancer after doxorubicin encapsulation inside liposomes using the pH gradient-driven method [207].

A DNA-based nanostructure with self-assembly and a pyramid cage composed of four oligonucleotides was introduced by Charoenphol and Bermudez [208]. DNA nanostructures allow therapeutic molecules to be encapsulated within their interior space, or intercalated along their double-helical edges, or incorporated as a part of the structure itself. AS1411 aptamers were conjugated to DNA pyramids, where the modified 3′-poly adenine RNA aptamer (AS1411) bound to its complementary 3′-poly thymine residue in DNA-based nanostructure. The aptamer-displaying pyramids were found to be significantly more resistant to nuclease degradation with enhanced intracellular uptake and they selectively inhibited the growth of cancer cells [208].

Another interesting DNA icosahedra nanostructure, loaded with doxorubicin for targeting MCF-7 breast cancer cells, was functionalized with anti-mucin1 (anti-MUC1) aptamers through cDNA strand conjugation. Aptamer-conjugated DNA icosahedra nanostructures showed an efficient and specific internalization for killing epithelial cancer cells [209].

A polyplex composed of cationic polymer polyethyleneimine (PEI) with plasmid DNA (pDNA) containing the firefly luciferase gene was prepared [210]. The polyplex can electrostatically conjugate anti-MUC1 aptamer on the surface to form the pDNA/PEI/MUC1 complex. This aptamer-guided polyplex showed higher gene expression in a mouse xenograft model of human lung cancer and was useful as a tumor-targeted gene delivery system with high transfection efficiency [210]. Zhao et al. tested a PEI-citrate nanocomplex with anaplastic lymphoma kinase (ALK) siRNA, functionalized with anti-CD30 aptamer via non-covalent bonds, to target human anaplastic large cell lymphoma (ALCL). This targeted nanocomplex specifically silenced *ALK* gene expression, leading to growth arrest and apoptosis [211]. The same nanocomplex formula was adapted by Subramanian et al. with an electrostatic EpCAM aptamer (EpApt) against breast cancer cell lines. This EpApt nanocomplex was able to target EpCAM tumor cells, deliver the siRNA, and silence the target gene [212].

Another targeted delivery system based on a dendrimer and a hybrid single-strand DNA-A9 PSMA (prostate-specific membrane antigen) RNA aptamer, followed by doxorubucin chelating, was an example of a base pairing cDNA-mediated dendrimer–aptamer conjugation. This nanocarrier reveals the promising possibility of this chemoimmuno therapeutic system against prostate cancer for in vivo and in vitro models [213]. Similarly, the base pairing cDNA-mediated dendrimer–aptamer conjugation strategy was also adapted in the conjugation of sgc8 to the three-armed Y-shaped dendritic DNA nanostructure. This formulation exerted strong toxicity for a human T-cell acute lymphoblastic leukemia cell line [214].

### 4.5. Avidin–Biotin Coupling

**Figure 30:**
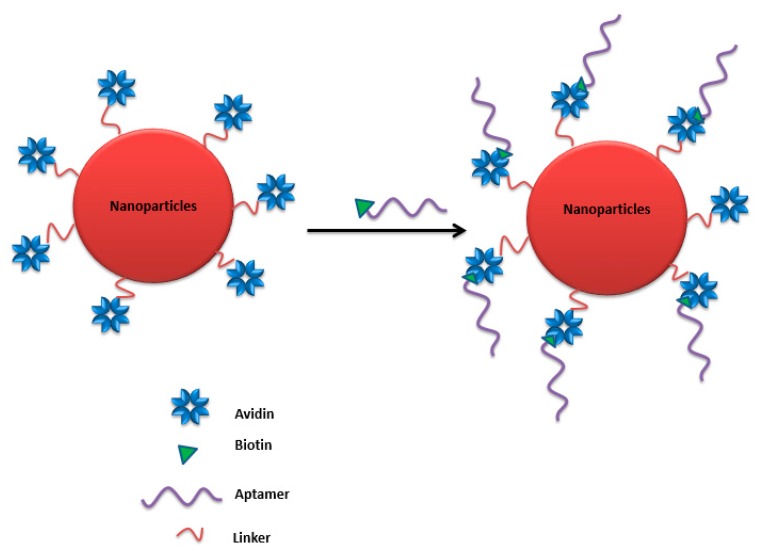
Avidin–biotin coupling, biotin attached theaptamer with avidin linked to the surface of the nanocarrier.

Avidin is a tetrameric biotin-binding protein. The tetrameric protein contains four identical subunits (homotetramer), each of which can bind to biotin (vitamin B7, vitamin H) with a high degree of affinity and specificity. The dissociation constant of the avidin–biotin complex was measured to be ≈ 10^−15^ M, making it one of the strongest known non-covalent bonds [215]. This binding approach was adapted by Ninomiya et al. to conjugate biotinylated aptamers on avidin-treated liposomes by avidin–biotin coupling (Figure 30), where an anti-platelet-derived growth factor receptor aptamer was surface-linked to doxorubicin-liposomes sensitized using poly (NIPMAM-co-NIPAM) as a thermosensitive polymer. Lower viability was observed against MDA-MB-231 breast cancer cell lines treated with doxorubicin-loaded aptamer-functionalized liposomes under ultrasound irradiation compared to cell viability without ultrasound irradiation [31].

Zhou et al. developed multifunctional aptamer-functionalized, doxorubicin-loaded calcium carbonate (CC) nanostructure platforms (Apt-CCNs), where a cross-linked avidin membrane on the surface of the CCNs was prepared to bind the biotin-modified sgc8 aptamer to prepare aptamer-modified and DOX-loaded CCNs. This nanostructure platform showed accurate cell targeting and controlled drug release [216].

An aptamer-conjugated Rubpy-doped silica nanoprobe has been tested for human breast carcinoma MCF-7 cells’ labeling. This nanoprobe was made by avidin–biotin coupling between 5′-biotin-labeled MUC-1 aptamer with avidin-functionalized silica NPs (avidin-FSiNPs) [189].

A biotin-modified Sgc8 aptamer was used to identify CCRF-CEM cells (a T-ALL cell line), and then biotin-appended QDs were labeled with the aptamer via streptavidin and biotin amplification interactions. The results revealed that the complex could be more effective in diagnosing leukemia at the early stage and has the potential to image tumor cells in vitro or in vivo for early diagnosis of disease [217].

### 4.6. Sulfhydryl-Aptamer Gold Coordination

**Figure 31:**
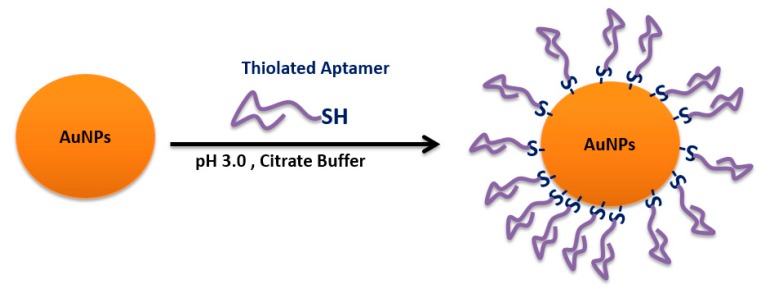
Coordination attachment of thiolated nucleic acids to gold nanoparticles (AuNPs).

The strength of the thiol–gold coordination interaction provides the basis to fabricate robust self-assembled monolayers for diverse applications [218]. The attachment of thiolated nucleic acids to gold nanoparticles (AuNPs) has enabled many milestone achievements in nanobiotechnology (Figure 31) [219]. AS1411 aptamer was conjugated to gold nanostars. To synthesize the AS1411-Au nanoconstructs, thiolated AS1411 was attached to the AuNS surface via the gold−sulfur bond in a 2-day “salt-aging” process [220]. The AS1411-Au nanoconstructs were tested on 12 cancer cell lines that represent four cancer subcategories, showing enhanced in vitro efficacy as a result of increased aptamer stability and high local concentrations of AS1411 [220].

Strong gold−thiol linkages were utilized to decorate gold nanoparticles (Au NPs) with AS1411 aptamer, which tethered with 21-base pairs of the (CGATCGA)3 sequence approached to the Au NPs. This new platform has been used as nanocarriers to co-deliver the photosensitizer, 5,10,15,20-tetrakis(1-methylpyridinium-4-yl) porphyrin (TMPyP4), and doxorubicin to target tumor cells, such as HeLa and Dox-resistant MCF-7R cell lines. The photodynamic stimulation of these platforms enhanced doxorubicin release in cancer cells with higher cytotoxicity compared to free doxorubicin [221].

5′-thiol-modified sgc8c aptamer selective to CCRF-CEM cells (T-cell acute lymphoblastic leukemia cell line) was tethered to hairpin DNA–gold nanoparticle conjugates through the gold–thiol linkage. The d(CGATCG) sequence within the hairpin DNA on the gold nanoparticle surface was used for doxorubicin intercalating. The constructed nanoconjugates accommodated a high drug loading and showed specific recognition of tumor cells in addition to triggered release of the encapsulated molecules when exposed to laser illumination [222].

3′-thiolated PTK7 aptamer (sgc8c)-functionalized gold nanoparticles, via sulfur-gold linkage, was synthesized to target T-cell acute lymphoblastic leukemia. Daunorubicin, an antitumor drug, was chelated into CG-rich sequences of sgc8c aptamer. The resulting Apt-daunorubicin-Au nanoparticles showed a higher internalization and delivery of drug into cells, with a better release in response to slightly acidic pH [30].

A photodynamic and photothermal cancer therapy multimodal composed of an aptamer switch probe (ASP) attached to a photosensitizer molecule, chlorin e6-polyvinylpyrrolidone (Ce6-PVP), was tested against different leukemia cell lines. The photosensitizer, Ce6, is connected to the 3′-end of ASP via coupling between the carboxyl group of the Ce6 molecule and the amino group at the 3′-end of the sgc8 aptamer. A poly-T chain links the 5′-end of sgc8 to an eight-base segment complementary to sgc8, which ends in a sulfhydryl group attachment to AuNRs. The AuNR-ASP-Ce6 composite enhanced targeted binding and provided high specificity and therapeutic efficiency [223].

Another efficient photothermal therapy based on aptamer-conjugated Au-Ag nanorods was tested on mixed cancer cells by Huang et al. The selected sgc8c aptamer, after 5′-thiol modification, was attached to the nanorods′ surfaces through simple thiol–Au linkage. The aptamer-functionalized nanorods killed 50% of the CCRF-CEMM cells compared to a 13% cell death in the control cells (NB-4) [224].

**Figure 32:**
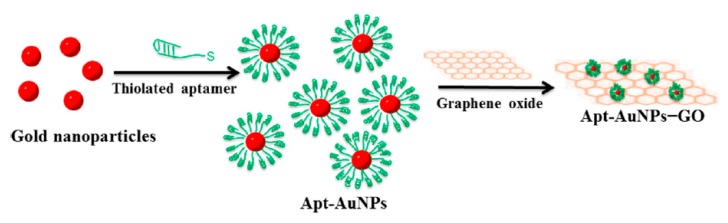
Schematic representation of the preparation of Apt-AuNP−GO [225].

A nanocomposite of aptamer−gold nanoparticle-hybridized graphene oxide (Apt-AuNP−GO) was designed as a photothermal treatment of MUC1-positive human breast cancer cells (MCF-7). The thiolated-muc1 aptamer was linked to the gold nanoparticle, via sulfur–gold linkage, to facilitate targeted treatment of the tumor cells (Figure 32). The Apt-AuNP−GO photothermal treatment led to targeted inhibition of breast cancer MCF-7 cells’ growth by inducing apoptosis [225].

Zhao et al. utilized this approach for the dual targeting Au shell-coated-liposomes with sulfhydryl anti-MUC1 aptamer (S2.2) and the AS1411 aptamer. The preformed liposomes were mixed with gold salt to form a uniformly distributed Au shell on the liposomes′ surfaces, followed by sulfhydryl-aptamer coordination on the gold nanoparticle. These liposomes loaded with docetaxel and ammonium chloride, with dual ligand functionalization, significantly increased cellular uptake in breast cancer cell line (MCF-7) cells and showed higher tumor suppression compared to single targeting by one aptamer [226].

### 4.7. Oxidative Coupling

**Figure 33:**
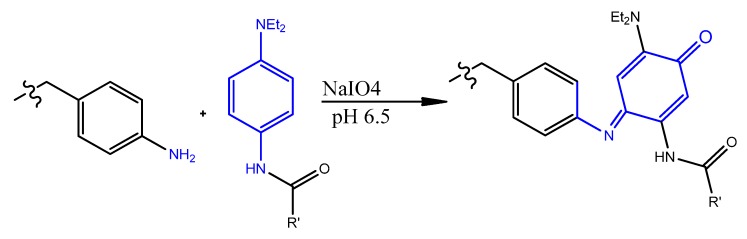
Oxidative coupling via a periodate-mediated reaction of phenylenediamine with aniline groups.

This conjugation strategy involves the periodate-mediated reaction of phenylene diamine-substituted aptamer, with aniline groups installed on the outer surface of the nanoparticle (Figure 33) [227].

**Figure 34:**
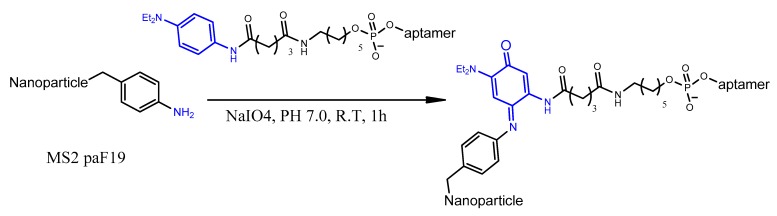
The MS2 surface was functionalized with sgc8c aptamer via oxidative coupling chemistry.

This approach is mainly applied to biomimetic nanocarriers. For example, bacteriophage MS2 virus has a protein coat of 180 sequence-identical monomers, expressed and self-assembled in *Escherichia coli*, that are arranged in a hollow spherical nanostructure [228]. This robust, safe, biodegradable, and genome-free nanostructure has many pores to encapsulate active medical ingredients in addition to the possibility of interior and exterior bioconjugation [29]. The MS2 interior was loaded with porphyrins capable of producing cytotoxic singlet oxygen upon illumination, then the surface was functionalized with sgc8c aptamer for targeting protein tyrosine kinase 7 (PTK7) receptors on Jurkat leukemia T cells. This method includes the chemo-selective coupling of an *N*,*N*-diethyl-*N*′-acylphenylene diamine moiety attached to the aptamer to an aniline residue on the MS2 capsid surface in the presence of sodium periodate (NaIO_4_). The aniline coupling partners can be introduced on the exterior surface of MS2 capsids either through direct chemical modification [227] or through the introduction of an unnatural amino acid, p-aminophenylalanine (paF), into position 19 of the MS2 coat protein using the amber stop codon suppression system (Figure 34) [229]. This targeted biomimetic nanocarrier selectively targets and kills 76% of the tumor cells after only 20 min of illumination [29].

### 4.8. Click Chemistry

**Figure 35:**
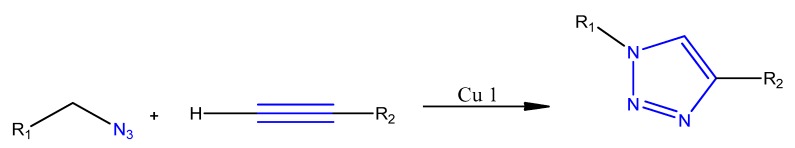
The general CuAAC reaction.

The copper-catalyzed azide–alkyne cycloaddition reaction is widely used for the connection of molecular entities of all sizes (Figure 35) [32].

**Figure 36:**
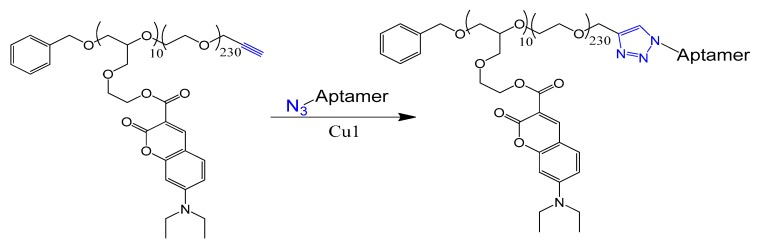
Functionalization of ω-alkyne-polyether to give aptamer-polymer through the CuAAC reaction.

Aptamer-polymer hybrids (APHs) were synthesized based on the coupling of the 3′-*N*_3_-AS1411 aptamer with a ω-alkyne-functionalized polyether by click chemistry using tricarboxylate ligand (BimC4A) to stabilize Cu (I) during the cycloaddition. APH molecules, being loaded with doxorubicin, are actively internalized via endocytosis into MCF-7 cells and selectively kill nucleolin-expressing target cells (Figure 36) [230].

Table 3 represents a summary of the various conjugation strategies of aptamers to different nanoparticles for drug targeting to cancer cells. 

## 5. Aptamer Toxicity and Immunogenicity

Although aptamers’ low toxicity and immunogenicity have been demonstrated by the majority of studies published in the literature, some reported cases indicated that administrated doses of aptamers were associated with a degree of toxicity and immunogenicity. As an example, macugen, an anti-VEGF aptamer, showed minimal toxicity and no serious drug-related side effects when injected as single or multiple doses into humans and animals [278,279,280,281]. The previously mentioned studies demonstrated the safety of macugen when locally administrated in the eye. Thus, the systemically administrated aptamers might not always show the same level of safety as macugen [282]. Furthermore, macugen is the only aptamer with proven clinical safety over an extended period of two years [283]. Aptamers tested in future clinical trials might reveal contradicting results. The polyanionic nature of aptamers may result in nonspecific interactions with human serum proteins. This could lead aptamers accumulating in tissues, causing specific cases, and serious, and possibly life-threatening, side effects [284,285]. The toxicity and immunogenicity of aptamers may also be the result of some chemical modifications. For example, phosphorothiolated aptamers were associated with activation of the complement system [286]. Pattern recognition receptors (PRRs) are a part of the innate immune system, which detects foreign nucleic acids. Aptamers containing CpG may resemble pathogen-associated molecular patterns (PAMPs), triggering the innate immune system and inducing the expression of deleterious cytokines [287,288]. Additionally, LNA aptamers impose a significant risk of hepatotoxicity [289,290]. Modifying aptamers with hydrophobic moieties increases their promiscuity, potentially increasing toxicity. Also, lipophilic particles have low hepatic clearance [291]. RNA aptamers modified with the 2′-fluoro group represent another chemical modification that might be a concern since these aptamers may activate PRRs. Although this may be advantageous in cancer therapies, it may also produce unwanted side effects. For example, 2′-fluoro pyrimidine-modified aptamers can enhance the activity of retinoic acid-inducible gene 1 (RIG-1), and increase apoptosis and interferon-β expression in human cancer cells. On the other hand, 2′O-methyl pyrimidines fail to induce such an immune response [292]. The immune system may produce antibodies against PEGylated aptamers. For example, a phase 2 trial evaluating pegnivacogin, a PEGylated RNA aptamer, reported an allergic reaction induced by the aptamer [293]. In conclusion, even though aptamers may not have performed as anticipated in some clinical trials, they are still considered a safer alternative for antibodies. Nonetheless, further extensive clinical evaluation of their toxicity and immunogenicity is highly recommended for the production of a safer aptamer generation.

## 6. Conclusions

Aptamers are specific nucleic acid-based binding ligands that have refined properties, making them good candidates for various biomedical applications at the diagnostic, therapeutic, and targeted drug delivery levels. Since the discovery of aptamers, several chemical modifications have been introduced to enhance their resistance against nucleases and improve their stability in vivo. As for targeting ligands, aptamers have been successfully conjugated to nanocarriers, drug molecules, and nucleic acids, and they have been successfully implicated in the selective targeting and delivery of therapeutic payloads into targeted cells. Currently, there are several aptamers in various stages of clinical trials, with many already showing promising preclinical data. The field of aptamers is relatively new, but there is a growing body of evidence that it will contribute significantly to the future advancement of the new generation of targeted therapeutics.

Aptamers stand for a remarkable new family of medicinal agents placed between conventional organic molecules and biological drugs. Aptamers have size advantages, can be easily chemically synthesized, are largely non-toxic and non-immunogenic, and have high affinities and specificities that are comparable to antibodies. Aptamers can be synthesized and selected to a range of targets more efficiently.

Despite all these advantages, aptamers suffer from decreased in vivo stability and high renal excretion. Chemical modifications of aptamers can enhance their pharmacokinetic properties and improve their bioavailability. Moreover, aptamer chemical or physical conjugation to different types of drugs or even to target nanoparticles can expand the range of their medical applications.

## Figures and Tables

**Table 1:** Different SELEX methods that have been developed since aptamer discovery.

SELEX Type	Description	Ref.
Metal-Dependant Aptamers	Enrichment of oligonucleotide library with and without ion salts to generate aptamers that only function in the presence of metal ion salts.	[34]
Crossover-SELEX	First, oligonucleotides are enriched using cell-SELEX. The product of cell-SELEX is then enriched against its purified protein to yield a higher binding affinity. Crossover-SELEX is useful for targets that are rare in their original environment.	[35]
Subtractive SELEX	Selection of aptamers that have the ability to differentiate between two closely related targets (e.g., distinguishing between a normal cell structure and another disease-related one). This is obtained by adding rounds of negative selection against normal cells.	[36]
Conditional SELEX	Selection of aptamers that are affected by the presence of regulatory molecules; aptamer selection is performed in two stages here: The first stage in the presence of regulatory molecules and the second in the absence of regulatory molecules. Only the sequences that successfully bind to the target in either one of the stages but not the other is selected, depending on whether the aptamer is to be used in the presence or absence of regulatory molecules.	[37]
On-chip selection	This is similar to the microarray method. Single and double base variations are introduced using in silico methods to a pre-selected sequence with the highest affinity to its target and then embedded on a surface plasmon resonance (SPR) chip. On-chip selection is useful for aptamer selection against a large number of targets.	[38]
Immobilization-free SELEX or GO-SELEX	First, the library is incubated with the target, and graphene oxide (GO) is then added to the mix in order to bind the unbound sequences via p–p stacking.	[39]
Tissue slide-based SELEX	Selection of aptamers against clinical samples. Cancerous tissue is used in the first stage as a target. Then, the tissue is scraped from the slide with the bound sequences. These sequences are then eluted, and counter selection against normal tissue is performed to eliminate shared aptamers.	[40]
Capillary Electrophoresis SELEX (CE-SELEX)	CE-SELEX separates the target bounded from unbounded sequences by the difference in electrophoretic mobility, which is a highly efficient separation method. This method enables the selection of aptamer candidates with high affinity while reducing the selection rounds to 1 to 4 from nearly 20 in conventional SELEX	[41,42]
Microfluidic SELEX (M-SELEX)	Combining traditional SELEX with a microfluidic system. This system contains reagent-loaded micro-lines, a pressurized reagent reservoir manifold, a PCR thermocycler, and actuatable valves for selection and sample routing.	[43]
High-Throughput Sequencing SELEX (HTS-SELEX)	Aptamers are identified through an iterative process of evolutionary selection starting from a random pool containing billions of sequences. The most predominant characteristic of HTS-SELEX is that it firstly allows for sequencing of the library across all the selection rounds. Thus, enriched sequences are visible at a much earlier round, which is more time efficient. Fewer selection rounds also avoid the potential PCR bias caused by over selection.	[44]

**Table 2:** Comparison of the major differences between aptamers and antibodies [5,48,49,50,51].

	Aptamers	Antibodies
Synthesis	Chemically synthesized and easy to produce	High cost and complexity of production
Size	Small compared to antibodies	Large
Stability	Prone to nuclease degradation	Short biological half-life
Targets	Wide range of targets, starting from ions to whole living cells	Produced only against immunogenic molecules, which limits the range of targets
Toxicity and Immunogenicity	Low toxicity and non-immunogenic	Immunogenic
Binding Specificity	High binding specificity	High binding specificity
Binding Affinity	High binding affinity	High binding affinity
Clearance Rate	Rapid circulation clearance	Low clearance rate
Chemical Conjugation	Easy to conjugate to nanoparticles and drugs	More difficult to conjugate
Chemical modification	Tolerant to chemical modifications to enhance structural and functional propertie	Modifications often lead to reduced activity

**Table 3:** A summary of the conjugation methods of aptamers to different nanoparticles.

Target	Aptamer	Nanoparticle	Drug/Imaging Molecule	Tumors	Conjugation Methodology	Ref.
Nucleolin	AS1411	PLGA-b-PEG	Paclitaxel	Glioma	Carbodiimide chemistry	[231]
		Polyvalent mesoporous nanoparticles	Doxorubicin	Breast	Thiol-maleimide chemistry	[232]
		pegylated PAMAM dendrimer	Camptothecin	Colorectal	Thiol-maleimide chemistry	[233]
		polydopamine were surface modify a PLGA-b-TPGS polymer	Docetaxel	Breast	Thiol-maleimide related chemistry/Michael addition on dihydroxyindole unit	[234]
		PLGA-b-PEG	Doxorubicin and superparamagnetic iron oxide	Glioma	Carbodiimide chemistry	[235]
		polymersome	Doxorubicin	Breast	3′-Cholesterol AS1411/direct conjugation	[236]
		PAMAM-PEG	5-fluorouracil	Gastric cancer	Thiol-maleimide chemistry	[237]
		Alkyl-modified PAMAM dendrimers	Bcl-xLshRNA	Lung Cancer	Carbodiimide chemistry	[238]
PSMA	A10 (F-RNA)	PEGylated liposomes	^225^Ac	Prostate	Carbodiimide chemistry	[239]
		PLGA-b-PEG	Cis-Pt(IV)	Prostate	Carbodiimide chemistry	[240]
		TCL-SPION	Doxorubicin	Prostate	Carbodiimide chemistry of oligonucleotide linker followed by aptamer complementary base pair binding	[241]
	A10-3-J1	Superparamagnetic iron oxide	Doxorubicin	Prostate	Avidin-biotin DNA linker followed by aptamer complementary base pair binding	[242]
	A10-3.2	Atelocollagen	miR-15a and miR-16-	Prostate	Thiol-maleimide chemistry	[243]
MUC1	DNA aptamer	CuInS2 quantum dot	Daunorubicin	Prostate	Carbodiimide chemistry of oligonucleotide linker followed by aptamer complementary base pair binding	[244]
MUC1	DNA aptamer	Zn-doped CdTe QDs	Zn^2+^ doped CdTe QDs	Lung	Complementary DNA	[245]
		iron oxide nanoparticles	Hyperthermia	Breast	Avidin-biotin coupling	[246]
		Chitosan-coated human serum albumin	Paclitaxel	Breast	Carbodiimide chemistry	[247]
		Poloxamer	miRNA-29b	Lung	Carbodiimide chemistry	[248]
		Au@SPIONs	Photothermal therapy	Colon	SH-Aptamer gold coordination	[249]
		Micelle	Doxorubicin and proapoptotic peptide (KLA)	Breast, Colon	Carbodiimide chemistry	[250]
	5TR1 DNA aptamer	PLGA modified with chitosan	Epirubicin	Breast	Electrostatic interaction	[251]
	DNA aptamer MA3	Thermosensitive hydrogel	Doxorubicin	Breast	Thiol-maleimide chemistry	[252]
PTK7	Sgc8 (DNA)	Polyvalent aptamer system	Doxorubicin	T-cell acute lymphoblastic leukaemia	Complementary DNA	[253]
		Au-Ag nanorods	Doxorubicin	T-cell acute lymphoblastic leukemia	SH-Aptamer gold coordination	[254]
		Single-walled carbon nanotubes	Daunorubicin	T-cell acute lymphoblastic leukemia	direct conjugation	[255]
PTK7	Sgc8 (DNA)	Mesoporus nanoparticles	Doxorubicin	T-cell acute lymphoblastic leukaemia	Carbodiimide chemistry	[256]
		Gold nanoparticles	Daunorubicin	T-cell acute lymphoblastic leukemia	SH-Aptamer gold coordination	[257]
		Au	Doxorubicin	T-cell acute lymphoblastic leukemia	SH-Aptamer gold coordination	[258]
		Acoustic droplets	Daunorubicin	T-cell acute lymphoblastic leukemia	Thiol-maleimide chemistry	[259]
IgM	TDO5 (DNA)	PAMAM Dendrimer	Uptake study	Burkitt’s lymphoma	Carbodiimide chemistry	[260]
HER2	S6 aptamer	Plasmonic gold coating on magnetic nanoparticles	Fe3O4	Breast	SH-Aptamer gold coordination	[261]
	TSA14	PEGylated Liposomes	Doxorubicin	Breast	Thiol-maleimide chemistry	[262]
	A6	hybrid nanoparticles (cationic lipids and PLGA-b-PEG)	siRNA	Breast	Thiol-maleimide chemistry	[263]
CD44	DNA thiolated aptamer	PEG-PAMAM	miRNA	Breast	Carbodiimide chemistry for PAMAM followed by Aptamer Thiol-maleimide chemistry	[264]
EpCAM	EpApt	PLGA-b-PEG	Lecithisn curcumin	Colorectal	Carbodiimide chemistry	[265]
	DNA-EpCAM	mesoporous silica	Doxorubicin	colon	Carbodiimide chemistry	[266]
EGFR	RNA	Lipid-polymer nanoparticle	Salinomycin	Osteosarcoma CSCs	Thiol-maleimide chemistry	[267]
Tenascin-C	GBI-10	PEGylated Liposomes	Gadolinium Compounds	Glioma	Carbodiimide chemistry	[268]
	GBI-10	QD–Apt nanoprobes	CdSe/ZnS	Glioma	Carbodiimide chemistry	[269]
PDGFR	Gint4.T	PLGA-b-PEG	PI3K-mTOR inhibitor	glioblastoma	Carbodiimide chemistry	[270]
Cell-SELEX	SRZ1	Cationic-liposomes	Doxorubicin	Breast cancer	Avidin-biotin coupling	[271]
fibronectin protein	DNA aptamer AS-14	gold-coated magnetic nanoparticles	Magnetodynamic nanotherapy	Ehrlich carcinoma	Thiol-maleimide chemistry	[272]
Cell-SELEX	KW16-13	gold nanorods	Photothermal therapy	Breast	Thiol-maleimide chemistry	[273]
FGFR1	DNA aptamer	Iron oxide nanoparticles	Hyperthermia	Osteosarcoma	Avidin-biotin coupling	[274]
Nucliolin MUC1 ATP	AS1411 MUC1 ATP	DNA dendrimers, pH sensitive release	Epirubicin	Breast, Colon	Electrostatic interaction	[275]
Annexin A2	Annexin A2 aptamer	DNA/RNA hybrid Nanoparticles	Doxorubicin	Ovarian cancer	Complementary base pairing	[276]
CD20	DNA aptamer	Lipid-polymer Nanoparticles	Salinomycin	Melanoma	Thiol-maleimide chemistry	[277]

## References

[1] Ni X., Castanares M., Mukherjee A., Lupold S.E. (2011). Nucleic acid aptamers: Clinical applications and promising new horizons. Curr. Med. Chem..

[2] Ellington A.D., Szostak J.W. (1990). In Vitro selection of RNA molecules that bind specific ligands. Nature.

[3] Wilbanks B., Smestad J., Heider R.M., Warrington A.E., Rodriguez M., Maher L.J. (2019). Optimization of a 40-mer Antimyelin DNA Aptamer Identifies a 20-mer with Enhanced Properties for Potential Multiple Sclerosis Therapy. Nucleic Acid Ther..

[4] Amato T., Virgilio A., Pirone L., Vellecco V., Bucci M., Pedone E., Esposito V., Galeone A. (2019). Investigating the properties of TBA variants with twin thrombin binding domains. Sci. Rep..

[5] Nimjee S.M., White R.R., Becker R.C., Sullenger B.A. (2017). Aptamers as Therapeutics. Annu. Rev. Pharmacol. Toxicol..

[6] Bahreyni A., Ramezani M., Alibolandi M., Hassanzadeh P., Abnous K., Taghdisi S.M. (2019). High affinity of AS1411 toward copper; its application in a sensitive aptasensor for copper detection. Anal. Biochem..

[7] Wu Y., Midinov B., White R.J. (2019). Electrochemical Aptamer-Based Sensor for Real-Time Monitoring of Insulin. ACS Sens..

[8] Zhao H., Ma C., Chen M. (2019). A novel fluorometric method for inorganic pyrophosphatase detection based on G-quadruplex-thioflavin T. Mol. Cell. Probes.

[9] Soto Rodriguez P.E.D., Nash V.I.C., Filice M., Ruiz-Cabello J. (2019). Chapter 6—Aptamer-Based Strategies for Diagnostics. Nucleic Acid Nanotheranostics.

[10] Platella C., Riccardi C., Montesarchio D., Roviello G.N., Musumeci D. (2017). G-quadruplex-based aptamers against protein targets in therapy and diagnostics. Biochim. Biophys. Acta Gen. Subj..

[11] Nimjee M.S., Rusconi C., Sullenger B.A. (2005). Aptamers: An emerging class of therapeutics. Annu. Rev. Med..

[12] Campbell M.A., Wengel J. (2011). Locked vs. unlocked nucleic acids (LNA vs. UNA): Contrasting structures work towards common therapeutic goals. Chem. Soc. Rev..

[13] Peng C.G., Damha M.J. (2007). G-quadruplex induced stabilization by 2′-deoxy-2′-fluoro-D-arabinonucleic acids (2′F-ANA). Nucleic Acids Res..

[14] Sacca B., Lacroix L., Mergny J.L. (2005). The effect of chemical modifications on the thermal stability of different G-quadruplex-forming oligonucleotides. Nucleic Acids Res..

[15] Zaitseva M., Kaluzhny D., Shchyolkina A., Borisova O., Smirnov I., Pozmogova G. (2010). Conformation and thermostability of oligonucleotide d(GGTTGGTGTGGTTGG) containing thiophosphoryl internucleotide bonds at different positions. Biophys. Chem..

[16] Dougan H., Lyster D.M., Vo C.V., Stafford A., Weitz J.I., Hobbs J.B. (2000). Extending the lifetime of anticoagulant oligodeoxynucleotide aptamers in blood. Nuclear Med. Biol..

[17] de Smidt P.C., Le Doan T., de Falco S., van Berkel T.J. (1991). Association of antisense oligonucleotides with lipoproteins prolongs the plasma half-life and modifies the tissue distribution. Nucleic Acids Res..

[18] Obika S., Nanbu D., Hari Y., Morio K.-I., In Y., Ishida T., Imanishi T. (1997). Synthesis of 2′-O,4′-C-methyleneuridine and -cytidine. Novel bicyclic nucleosides having a fixed C3, -endo sugar puckering. Tetrahedron Lett..

[19] Koshkin A.A., Singh S.K., Nielsen P., Rajwanshi V.K., Kumar R., Meldgaard M., Olsen C.E., Wengel J. (1998). LNA (Locked Nucleic Acids): Synthesis of the adenine, cytosine, guanine 5-methylcytosine, thymine and uracil bicyclonucleoside monomers, oligomerisation, and unprecedented nucleic acid recognition. Tetrahedron.

[20] Dong H., Han L., Wang J., Xie J., Gao Y., Xie F., Jia L. (2018). In Vivo inhibition of circulating tumor cells by two apoptosis-promoting circular aptamers with enhanced specificity. J. Control Release.

[21] Kang Y.Y., Song J., Jung H.S., Kwak G., Yu G., Ahn J.-H., Kim S.H., Mok H. (2018). Implication of multivalent aptamers in DNA and DNA–RNA hybrid structures for efficient drug delivery in vitro and in vivo. J. Ind. Eng. Chem..

[22] Bouhedda F., Fam K.T., Collot M., Autour A., Marzi S., Klymchenko A., Ryckelynck M. (2019). A dimerization-based fluorogenic dye-aptamer module for RNA imaging in live cells. Nat. Chem. Biol..

[23] Padilla R., Sousa R. (1999). Efficient synthesis of nucleic acids heavily modified with non-canonical ribose 2′-groups using a mutantT7 RNA polymerase (RNAP). Nucleic Acids Res..

[24] Maio G., Enweronye O., Zumrut H.E., Batool S., Van N., Mallikaratchy P. (2017). Systematic optimization and modification of a DNA aptamer with 2′-O-methyl RNA analogues. ChemistrySelect.

[25] Carvalho J., Lopes-Nunes J., Lopes A.C., Cabral Campello M.P., Paulo A., Queiroz J.A., Cruz C. (2019). Aptamer-guided acridine derivatives for cervical cancer. Org. Biomol. Chem..

[26] Wen S., Miao X., Fan G.-C., Xu T., Jiang L.-P., Wu P., Cai C., Zhu J.-J. (2019). Aptamer-Conjugated Au Nanocage/SiO_2_ Core–Shell Bifunctional Nanoprobes with High Stability and Biocompatibility for Cellular SERS Imaging and Near-Infrared Photothermal Therapy. ACS Sens..

[27] Martínez-Jothar L., Doulkeridou S., Schiffelers R.M., Sastre Torano J., Oliveira S., van Nostrum C.F., Hennink W.E. (2018). Insights into maleimide-thiol conjugation chemistry: Conditions for efficient surface functionalization of nanoparticles for receptor targeting. J. Control. Release.

[28] Savla R., Taratula O., Garbuzenko O., Minko T. (2011). Tumor targeted quantum dot-mucin 1 aptamer-doxorubicin conjugate for imaging and treatment of cancer. J. Control. Release.

[29] Stephanopoulos N., Tong G.J., Hsiao S.C., Francis M.B. (2010). Dual-surface modified virus capsids for targeted delivery of photodynamic agents to cancer cells. ACS Nano.

[30] Danesh N.M., Lavaee P., Ramezani M., Abnous K., Taghdisi S.M. (2015). Targeted and controlled release delivery of daunorubicin to T-cell acute lymphoblastic leukemia by aptamer-modified gold nanoparticles. Int. J. Pharm..

[31] Ninomiya K., Yamashita T., Kawabata S., Shimizu N. (2014). Targeted and ultrasound-triggered drug delivery using liposomes co-modified with cancer cell-targeting aptamers and a thermosensitive polymer. Ultrason Sonochem..

[32] Presolski I.S., Hong V., Finn M.G. (2011). Copper-Catalyzed Azide-Alkyne Click Chemistry for Bioconjugation. Curr. Protoc. Chem. Biol..

[33] Kaur H. (2018). Recent developments in cell-SELEX technology for aptamer selection. Biochim. Biophys. Acta Gen. Subj..

[34] Kawakami J., Imanaka H., Yokota Y., Sugimoto N. (2000). In Vitro selection of aptamers that act with Zn^2+^. J. Inorg. Biochem..

[35] Hicke B.J., Marion C., Chang Y.F., Gould T., Lynott C.K., Parma D., Schmidt P.G., Warren S. (2001). Tenascin-C aptamers are generated using tumor cells and purified protein. J. Biol. Chem..

[36] Wang C., Zhang M., Yang G., Zhang D., Ding H., Wang H., Fan M., Shen B., Shao N. (2003). Single-stranded DNA aptamers that bind differentiated but not parental cells: Subtractive systematic evolution of ligands by exponential enrichment. J. Biotechnol..

[37] Darmostuk M., Rimpelova S., Gbelcova H., Ruml T. (2015). Current approaches in SELEX: An update to aptamer selection technology. Biotechnol. Adv..

[38] Asai R., Nishimura S.I., Aita T., Takahashi K. (2004). In Vitro Selection of DNA Aptamers on Chips Using a Method for Generating Point Mutations. Anal. Lett..

[39] Park J.-W., Tatavarty R., Kim D.W., Jung H.-T., Gu M.B. (2012). Immobilization-free screening of aptamers assisted by graphene oxide. Chem. Commun..

[40] Li S.H., Ding H., Huang Y., Cao X., Yang G., Li J., Xie Z., Meng Y., Li X., Zhao Q. (2009). Identification of an aptamer targeting hnRNP A1 by tissue slide-based SELEX. J. Pathol..

[41] Dong L., Tan Q., Ye W., Liu D., Chen H., Hu H., Wen D., Liu Y., Cao Y., Kang J. (2015). Screening and Identifying a Novel ssDNA Aptamer against Alpha-fetoprotein Using CE-SELEX. Sci. Rep..

[42] Hamedani N.S., Muller J. (2016). Capillary Electrophoresis for the Selection of DNA Aptamers Recognizing Activated Protein C. Methods Mol. Biol..

[43] Hybarger G., Bynum J., Williams R.F., Valdes J.J., Chambers J.P. (2006). A microfluidic SELEX prototype. Anal. Bioanal. Chem..

[44] Quang N.N., Perret G., Duconge F. (2016). Applications of High-Throughput Sequencing for in vitro Selection and Characterization of Aptamers. Pharmaceuticals.

[45] Wang H., Zhang Y., Yang H., Qin M., Ding X., Liu R., Jiang Y. (2018). In Vivo SELEX of an Inhibitory NSCLC-Specific RNA Aptamer from PEGylated RNA Library. Mol. Ther. Nucleic Acids.

[46] Bel N., Das A.T., Berkhout B. (2014). In Vivo SELEX of single-stranded domains in the HIV-1 leader RNA. J. Virol..

[47] Cheng C., Chen Y.H., Lennox K.A., Behlke M.A., Davidson B.L. (2013). In Vivo SELEX for Identification of Brain-penetrating Aptamers. Mol. Ther. Nucleic Acids.

[48] Zhang Y., Lai B.S., Juhas M. (2019). Recent Advances in Aptamer Discovery and Applications. Molecules.

[49] Alshaer W., Hillaireau H., Fattal E. (2018). Aptamer-guided nanomedicines for anticancer drug delivery. Adv. Drug Deliv. Rev..

[50] Thiviyanathan V., Gorenstein D.G. (2012). Aptamers and the next generation of diagnostic reagents. Proteom. Clin. Appl..

[51] Ulrich H., Trujillo C.A., Nery A.A., Alves J.M., Majumder P., Resende R.R., Martins A.H. (2006). DNA and RNA aptamers: From tools for basic research towards therapeutic applications. Comb. Chem. High. Throughput Screen.

[52] Diafa S., Hollenstein M. (2015). Generation of Aptamers with an Expanded Chemical Repertoire. Molecules.

[53] Kratschmer C., Levy M. (2017). Effect of Chemical Modifications on Aptamer Stability in Serum. Nucleic Acid Ther..

[54] Tolle F., Mayer G. (2013). Dressed for success—applying chemistry to modulate aptamer functionality. Chem. Sci..

[55] Jellinek D., Green L.S., Bell C., Lynott C.K., Gill N., Vargeese C., Kirschenheuter G., McGee D.P., Abesinghe P., Pieken W.A. (1995). Potent 2′-amino-2′-deoxypyrimidine RNA inhibitors of basic fibroblast growth factor. Biochemistry.

[56] Kuwahara M., Sugimoto N. (2010). Molecular evolution of functional nucleic acids with chemical modifications. Molecules.

[57] Shum K.T., Tanner J.A. (2008). Differential inhibitory activities and stabilisation of DNA aptamers against the SARS coronavirus helicase. Chembiochem.

[58] Caruthers M.H., Barone A.D., Beaucage S.L., Dodds D.R., Fisher E.F., McBride L.J., Matteucci M., Stabinsky Z., Tang J.Y. (1987). Chemical synthesis of deoxyoligonucleotides by the phosphoramidite method. Methods Enzymol..

[59] Riccardi C., Russo Krauss I., Musumeci D., Morvan F., Meyer A., Vasseur J.J., Paduano L., Montesarchio D. (2017). Fluorescent Thrombin Binding Aptamer-Tagged Nanoparticles for an Efficient and Reversible Control of Thrombin Activity. ACS Appl. Mater. Interfaces.

[60] Ortigao J.F.R., ÖSch H., Montenarh M., FrÖHlich A., Seliger H. (1991). Oligonucleotide Analogs with Terminal 3′,3′- and 5′,5′-Internucleotidic Linkages as Antisense Inhibitors of Viral Replication. Antisense Res. Dev..

[61] Healy J.M., Lewis S.D., Kurz M., Boomer R.M., Thompson K.M., Wilson C., McCauley T.G. (2004). Pharmacokinetics and biodistribution of novel aptamer compositions. Pharm. Res..

[62] Prodeus A., Abdul-Wahid A., Fischer N.W., Huang E.H., Cydzik M., Gariepy J. (2015). Targeting the PD-1/PD-L1 Immune Evasion Axis With DNA Aptamers as a Novel Therapeutic Strategy for the Treatment of Disseminated Cancers. Mol. Ther. Nucleic Acids.

[63] Lee C.H., Lee S.H., Kim J.H., Noh Y.H., Noh G.J., Lee S.W. (2015). Pharmacokinetics of a Cholesterol-conjugated Aptamer Against the Hepatitis C Virus (HCV) NS5B Protein. Mol. Ther. Nucleic Acids.

[64] Willis M.C., Collins B.D., Zhang T., Green L.S., Sebesta D.P., Bell C., Kellogg E., Gill S.C., Magallanez A., Knauer S. (1998). Liposome-anchored vascular endothelial growth factor aptamers. Bioconjug. Chem..

[65] Riccardi C., Musumeci D., Russo Krauss I., Piccolo M., Irace C. (2018). Exploring the conformational behaviour and aggregation properties of lipid-conjugated AS1411 aptamers. Int. J. Biol. Macromol..

[66] Veronese F.M., Pasut G. (2005). PEGylation, successful approach to drug delivery. Drug Discov. Today.

[67] Hoffmann S., Hoos J., Klussmann S., Vonhoff S. (2011). RNA Aptamers and Spiegelmers: Synthesis, Purification, and Post-Synthetic PEG Conjugation. Curr. Protoc. Nucleic Acid Chem..

[68] Kazuhiko H., Natsuki O., Masakazu N., Tomoyoshi K., Asako S., Shinsuke H., Masayuki T., Kuniyoshi H., Hideaki S., Hiroaki Y. (2017). A Novel PEGylation Method for Improving the Pharmacokinetic Properties of Anti-Interleukin-17A RNA Aptamers. Nucleic Acid Ther..

[69] Da Pieve C., Blackshaw E., Missailidis S., Perkins A.C. (2012). PEGylation and biodistribution of an anti-MUC1 aptamer in MCF-7 tumor-bearing mice. Bioconjug. Chem..

[70] Tan L., Neoh K.G., Kang E.T., Choe W.S., Su X. (2011). PEGylated anti-MUC1 aptamer-doxorubicin complex for targeted drug delivery to MCF7 breast cancer cells. Macromol. Biosci..

[71] Ruckman J., Green L.S., Beeson J., Waugh S., Gillette W.L., Henninger D.D., Claesson-Welsh L., Janjic N. (1998). 2′-Fluoropyrimidine RNA-based aptamers to the 165-amino acid form of vascular endothelial growth factor (VEGF165). Inhibition of receptor binding and VEGF-induced vascular permeability through interactions requiring the exon 7-encoded domain. J. Biol. Chem..

[72] Lin Y., Qiu Q., Gill S.C., Jayasena S.D. (1994). Modified RNA sequence pools for in vitro selection. Nucleic Acids Res..

[73] Green L.S., Jellinek D., Bell C., Beebe L.A., Feistner B.D., Gill S.C., Jucker F.M., Janjic N. (1995). Nuclease-resistant nucleic acid ligands to vascular permeability factor/vascular endothelial growth factor. Chem. Biol..

[74] Pagratis N.C., Bell C., Chang Y.F., Jennings S., Fitzwater T., Jellinek D., Dang C. (1997). Potent 2′-amino-, and 2′-fluoro-2′-deoxyribonucleotide RNA inhibitors of keratinocyte growth factor. Nat. Biotechnol..

[75] Ng E.W., Shima D.T., Calias P., Cunningham E.T., Guyer D.R., Adamis A.P. (2006). Pegaptanib, a targeted anti-VEGF aptamer for ocular vascular disease. Nat. Rev. Drug Discov..

[76] Li N., Nguyen H.H., Byrom M., Ellington A.D. (2011). Inhibition of cell proliferation by an anti-EGFR aptamer. PLoS ONE.

[77] Esposito C.L., Passaro D., Longobardo I., Condorelli G., Marotta P., Affuso A., de Franciscis V., Cerchia L. (2011). A neutralizing RNA aptamer against EGFR causes selective apoptotic cell death. PLoS ONE.

[78] Svobodova M., Bunka D.H., Nadal P., Stockley P.G., O’Sullivan C.K. (2013). Selection of 2′F-modified RNA aptamers against prostate-specific antigen and their evaluation for diagnostic and therapeutic applications. Anal. Bioanal. Chem..

[79] Lupold S.E., Hicke B.J., Lin Y., Coffey D.S. (2002). Identification and characterization of nuclease-stabilized RNA molecules that bind human prostate cancer cells via the prostate-specific membrane antigen. Cancer Res..

[80] Thirunavukarasu D., Chen T., Liu Z., Hongdilokkul N., Romesberg F.E. (2017). Selection of 2′-Fluoro-Modified Aptamers with Optimized Properties. J. Am. Chem. Soc..

[81] Lauridsen H.L., Rothnagel J.A., Veedu R.N. (2012). Enzymatic Recognition of 2′-Modified Ribonucleoside 5′-Triphosphates: Towards the Evolution of Versatile Aptamers. ChemBioChem.

[82] Waters E.K., Genga R.M., Schwartz M.C., Nelson J.A., Schaub R.G., Olson K.A., Kurz J.C., McGinness K.E. (2011). Aptamer ARC19499 mediates a procoagulant hemostatic effect by inhibiting tissue factor pathway inhibitor. Blood.

[83] Xiao Z., Levy-Nissenbaum E., Alexis F., Luptak A., Teply B.A., Chan J.M., Shi J., Digga E., Cheng J., Langer R. (2012). Engineering of targeted nanoparticles for cancer therapy using internalizing aptamers isolated by cell-uptake selection. ACS Nano.

[84] Wilds C.J., Damha M.J. (2000). 2′-Deoxy-2′-fluoro-beta-D-arabinonucleosides and oligonucleotides (2′F-ANA): Synthesis and physicochemical studies. Nucleic Acids Res..

[85] Watts J.K., Katolik A., Viladoms J., Damha M.J. (2009). Studies on the hydrolytic stability of 2′-fluoroarabinonucleic acid (2′F-ANA). Org. Biomol. Chem..

[86] Watts J.K., Katolik A., Viladoms J., Damha M.J. (2010). Differential stability of 2′F-ANA*RNA and ANA*RNA hybrid duplexes: Roles of structure, pseudohydrogen bonding, hydration, ion uptake and flexibility. Nucleic Acids Res..

[87] Minakawa N., Sanji M., Kato Y., Matsuda A. (2008). Investigations toward the selection of fully-modified 4′-thioRNA aptamers: Optimization of in vitro transcription steps in the presence of 4′-thioNTPs. Bioorg. Med. Chem..

[88] Kato Y., Minakawa N., Komatsu Y., Kamiya H., Ogawa N., Harashima H., Matsuda A. (2005). New NTP analogs: The synthesis of 4′-thioUTP and 4′-thioCTP and their utility for SELEX. Nucleic Acids Res..

[89] Darfeuille F., Hansen J.B., Orum H., Di Primo C., Toulme J.J. (2004). LNA/DNA chimeric oligomers mimic RNA aptamers targeted to the TAR RNA element of HIV-1. Nucleic Acids Res..

[90] Shi H., He X., Cui W., Wang K., Deng K., Li D., Xu F. (2014). Locked nucleic acid/DNA chimeric aptamer probe for tumor diagnosis with improved serum stability and extended imaging window in vivo. Anal. Chim. Acta.

[91] Schmidt K.S., Borkowski S., Kurreck J., Stephens A.W., Bald R., Hecht M., Friebe M., Dinkelborg L., Erdmann V.A. (2004). Application of locked nucleic acids to improve aptamer in vivo stability and targeting function. Nucleic Acids Res..

[92] Hernandez F.J., Kalra N., Wengel J., Vester B. (2009). Aptamers as a model for functional evaluation of LNA and 2′-amino LNA. Bioorg. Med. Chem. Lett..

[93] Pasternak A., Hernandez F.J., Rasmussen L.M., Vester B., Wengel J. (2011). Improved thrombin binding aptamer by incorporation of a single unlocked nucleic acid monomer. Nucleic Acids Res..

[94] Veedu N.R., Vester B., Wengel J. (2009). Efficient enzymatic synthesis of LNA-modified DNA duplexes using KOD DNA polymerase. Org. Biomol. Chem..

[95] Kotkowiak W., Wengel J., Scotton C.J., Pasternak A. (2019). Improved RE31 Analogues Containing Modified Nucleic Acid Monomers: Thermodynamic, Structural, and Biological Effects. J. Med. Chem..

[96] Pozmogova G.E., Zaitseva M.A., Smirnov I.P., Shvachko A.G., Murina M.A., Sergeenko V.I. (2010). Anticoagulant effects of thioanalogs of thrombin-binding DNA-aptamer and their stability in the plasma. Bull. Exp. Biol. Med..

[97] Abeydeera N.D., Egli M., Cox N., Mercier K., Conde J.N., Pallan P.S., Mizurini D.M., Sierant M., Hibti F.E., Hassell T. (2016). Evoking picomolar binding in RNA by a single phosphorodithioate linkage. Nucleic Acids Res..

[98] Mann A.P., Somasunderam A., Nieves-Alicea R., Li X., Hu A., Sood A.K., Ferrari M., Gorenstein D.G., Tanaka T. (2010). Identification of thioaptamer ligand against E-selectin: Potential application for inflamed vasculature targeting. PLoS ONE.

[99] King D.J., Bassett S.E., Li X., Fennewald S.A., Herzog N.K., Luxon B.A., Shope R., Gorenstein D.G. (2002). Combinatorial selection and binding of phosphorothioate aptamers targeting human NF-kappa B RelA(p65) and p50. Biochemistry.

[100] Somasunderam A., Ferguson M.R., Rojo D.R., Thiviyanathan V., Li X., O’Brien W.A., Gorenstein D.G. (2005). Combinatorial selection, inhibition, and antiviral activity of DNA thioaptamers targeting the RNase H domain of HIV-1 reverse transcriptase. Biochemistry.

[101] Somasunderam A., Thiviyanathan V., Tanaka T., Li X., Neerathilingam M., Lokesh G.L., Mann A., Peng Y., Ferrari M., Klostergaard J. (2010). Combinatorial selection of DNA thioaptamers targeted to the HA binding domain of human CD44. Biochemistry.

[102] Gandham S.H., Volk D.E., Lokesh G.L., Neerathilingam M., Gorenstein D.G. (2014). Thioaptamers targeting dengue virus type-2 envelope protein domain III. Biochem. Biophys. Res. Commun..

[103] Leonard F., Ha N.P., Sule P., Alexander J.F., Volk D.E., Lokesh G.L.R., Liu X., Cirillo J.D., Gorenstein D.G., Yuan J. (2017). Thioaptamer targeted discoidal microparticles increase self immunity and reduce Mycobacterium tuberculosis burden in mice. J. Control. Release.

[104] Xianbin Y., David G.G. (2004). Progress in Thioaptamer Development. Curr. Drug Targets.

[105] Lokesh G.L., Wang H., Lam C.H., Thiviyanathan V., Ward N., Gorenstein D.G., Volk D.E. (2017). X-Aptamer Selection and Validation. Methods Mol. Biol..

[106] He W., Elizondo-Riojas M.A., Li X., Lokesh G.L., Somasunderam A., Thiviyanathan V., Volk D.E., Durland R.H., Englehardt J., Cavasotto C.N. (2012). X-aptamers: A bead-based selection method for random incorporation of druglike moieties onto next-generation aptamers for enhanced binding. Biochemistry.

[107] Prater C.E., Miller P.S. (2004). 3′-Methylphosphonate-Modified Oligo-2′-O-methylribonucleotides and Their Tat Peptide Conjugates:  Uptake and Stability in Mouse Fibroblasts in Culture. Bioconjugate Chem..

[108] Kibler-Herzog L., Zon G., Uznanski B., Whittier G., Wilson W.D. (1991). Duplex stabilities of phosphorothioate, methylphosphonate, and RNA analogs of two DNA 14-mers. Nucleic Acids Res..

[109] Mutisya D., Selvam C., Kennedy S.D., Rozners E. (2011). Synthesis and properties of triazole-linked RNA. Bioorg. Med. Chem. Lett.

[110] Sau S., Hrdlicka P.J. (2012). C2′-pyrene-functionalized triazole-linked DNA: Universal DNA/RNA hybridization probes. J. Org. Chem..

[111] Varizhuk A.M., Kaluzhny D.N., Novikov R.A., Chizhov A.O., Smirnov I.P., Chuvilin A.N., Tatarinova O.N., Fisunov G.Y., Pozmogova G.E., Florentiev V.L. (2013). Synthesis of triazole-linked oligonucleotides with high affinity to DNA complements and an analysis of their compatibility with biosystems. J. Org. Chem..

[112] El-Sagheer A.H., Brown T. (2010). Click chemistry with DNA. Chem. Soc. Rev..

[113] El-Sagheer A.H., Brown T. (2012). Click nucleic acid ligation: Applications in biology and nanotechnology. Acc. Chem. Res..

[114] Chandrasekhar S., Srihari P., Nagesh C., Kiranmai N., Nagesh N., Idris M.M. (2010). Synthesis of Readily Accessible Triazole-Linked Dimer Deoxynucleoside Phosphoramidite for Solid-Phase Oligonucleotide Synthesis. Synthesis.

[115] Varizhuk A.M., Tsvetkov V.B., Tatarinova O.N., Kaluzhny D.N., Florentiev V.L., Timofeev E.N., Shchyolkina A.K., Borisova O.F., Smirnov I.P., Grokhovsky S.L. (2013). Synthesis, characterization and in vitro activity of thrombin-binding DNA aptamers with triazole internucleotide linkages. Eur. J. Med. Chem..

[116] Hocek M. (2014). Synthesis of base-modified 2′-deoxyribonucleoside triphosphates and their use in enzymatic synthesis of modified DNA for applications in bioanalysis and chemical biology. J. Org. Chem..

[117] Hollenstein M. (2012). Synthesis of Deoxynucleoside Triphosphates that Include Proline, Urea, or Sulfonamide Groups and Their Polymerase Incorporation into DNA. Chem. A Eur. J..

[118] Latham A.J., Johnson R., Toole J.J. (1994). The application of a modified nucleotide in aptamer selection: Novel thrombin aptamers containing 5-(1-pentynyl)-2′-deoxyuridine. Nucleic Acids Res..

[119] Gupta S., Drolet D.W., Wolk S.K., Waugh S.M., Rohloff J.C., Carter J.D., Mayfield W.S., Otis M.R., Fowler C.R., Suzuki T. (2017). Pharmacokinetic Properties of DNA Aptamers with Base Modifications. Nucleic Acid Ther..

[120] Jensen K.B., Atkinson B.L., Willis M.C., Koch T.H., Gold L. (1995). Using in vitro selection to direct the covalent attachment of human immunodeficiency virus type 1 Rev protein to high-affinity RNA ligands. Proc. Natl. Acad. Sci. USA.

[121] Li M., Lin N., Huang Z., Du L., Altier C., Fang H., Wang B. (2008). Selecting aptamers for a glycoprotein through the incorporation of the boronic acid moiety. J. Am. Chem. Soc..

[122] Imaizumi Y., Kasahara Y., Fujita H., Kitadume S., Ozaki H., Endoh T., Kuwahara M., Sugimoto N. (2013). Efficacy of base-modification on target binding of small molecule DNA aptamers. J. Am. Chem. Soc..

[123] Shoji A., Kuwahara M., Ozaki H., Sawai H. (2007). Modified DNA aptamer that binds the (R)-isomer of a thalidomide derivative with high enantioselectivity. J. Am. Chem. Soc..

[124] Ohsawa K., Kasamatsu T., Nagashima J., Hanawa K., Kuwahara M., Ozaki H., Sawai H. (2008). Arginine-modified DNA aptamers that show enantioselective recognition of the dicarboxylic acid moiety of glutamic acid. Anal. Sci..

[125] MacPherson I.S., Temme J.S., Habeshian S., Felczak K., Pankiewicz K., Hedstrom L., Krauss I.J. (2011). Multivalent glycocluster design through directed evolution. Angew. Chem. Int. Ed. Engl..

[126] Temme J.S., Drzyzga M.G., MacPherson I.S., Krauss I.J. (2013). Directed evolution of 2G12-targeted nonamannose glycoclusters by SELMA. Chemistry.

[127] Temme J.S., MacPherson I.S., DeCourcey J.F., Krauss I.J. (2014). High temperature SELMA: Evolution of DNA-supported oligomannose clusters which are tightly recognized by HIV bnAb 2G12. J. Am. Chem. Soc..

[128] Lee K.Y., Kang H., Ryu S.H., Lee D.S., Lee J.H., Kim S. (2010). Bioimaging of nucleolin aptamer-containing 5-(N-benzylcarboxyamide)-2′-deoxyuridine more capable of specific binding to targets in cancer cells. J. Biomed. Biotechnol..

[129] Kimoto M., Yamashige R., Matsunaga K., Yokoyama S., Hirao I. (2013). Generation of high-affinity DNA aptamers using an expanded genetic alphabet. Nat. Biotechnol..

[130] Sefah K., Yang Z., Bradley K.M., Hoshika S., Jimenez E., Zhang L., Zhu G., Shanker S., Yu F., Turek D. (2014). In Vitro selection with artificial expanded genetic information systems. Proc. Natl. Acad. Sci. USA.

[131] Betz K., Kimoto M., Diederichs K., Hirao I., Marx A. (2017). Structural Basis for Expansion of the Genetic Alphabet with an Artificial Nucleobase Pair. Angew. Chem. Int. Ed..

[132] Renders M., Miller E., Lam C.H., Perrin D.M. (2017). Whole cell-SELEX of aptamers with a tyrosine-like side chain against live bacteria. Org. Biomol. Chem..

[133] Balintová J., Simonova A., Białek-Pietras M., Olejniczak A., Lesnikowski Z.J., Hocek M. (2017). Carborane-linked 2′-deoxyuridine 5′-O-triphosphate as building block for polymerase synthesis of carborane-modified DNA. Bioorg. Med. Chem. Lett..

[134] Vaught J.D., Bock C., Carter J., Fitzwater T., Otis M., Schneider D., Rolando J., Waugh S., Wilcox S.K., Eaton B.E. (2010). Expanding the Chemistry of DNA for in vitro Selection. J. Am. Chem. Soc..

[135] Pfeiffer F., Tolle F., Rosenthal M., Brandle G.M., Ewers J., Mayer G. (2018). Identification and characterization of nucleobase-modified aptamers by click-SELEX. Nat. Protoc..

[136] Gupta S., Hirota M., Waugh S.M., Murakami I., Suzuki T., Muraguchi M., Shibamori M., Ishikawa Y., Jarvis T.C., Carter J.D. (2014). Chemically modified DNA aptamers bind interleukin-6 with high affinity and inhibit signaling by blocking its interaction with interleukin-6 receptor. J. Biol. Chem..

[137] Hopfield J.J. (1974). Kinetic proofreading: A new mechanism for reducing errors in biosynthetic processes requiring high specificity. Proc. Natl. Acad. Sci. USA.

[138] Hathout Y., Brody E., Clemens P.R., Cripe L., DeLisle R.K., Furlong P., Gordish-Dressman H., Hache L., Henricson E., Hoffman E.P. (2015). Large-scale serum protein biomarker discovery in Duchenne muscular dystrophy. Proc. Natl. Acad. Sci. USA.

[139] Rohloff J.C., Gelinas A.D., Jarvis T.C., Ochsner U.A., Schneider D.J., Gold L., Janjic N. (2014). Nucleic Acid Ligands With Protein-like Side Chains: Modified Aptamers and Their Use as Diagnostic and Therapeutic Agents. Mol. Ther. Nucleic Acids.

[140] Duo J., Chiriac C., Huang R.Y.C., Mehl J., Chen G., Tymiak A., Sabbatini P., Pillutla R., Zhang Y. (2018). Slow Off-Rate Modified Aptamer (SOMAmer) as a Novel Reagent in Immunoassay Development for Accurate Soluble Glypican-3 Quantification in Clinical Samples. Anal. Chem..

[141] Gawande B.N., Rohloff J.C., Carter J.D., von Carlowitz I., Zhang C., Schneider D.J., Janjic N. (2017). Selection of DNA aptamers with two modified bases. Proc. Natl. Acad. Sci. USA.

[142] Wang Y., Ng N., Liu E., Lam C.H., Perrin D.M. (2017). Systematic study of constraints imposed by modified nucleoside triphosphates with protein-like side chains for use in in vitro selection. Org. Biomol. Chem..

[143] Ni S., Yao H., Wang L., Lu J., Jiang F., Lu A., Zhang G. (2017). Chemical Modifications of Nucleic Acid Aptamers for Therapeutic Purposes. Int. J. Mol. Sci..

[144] Hoellenriegel J., Zboralski D., Maasch C., Rosin N.Y., Wierda W.G., Keating M.J., Kruschinski A., Burger J.A. (2014). The Spiegelmer NOX-A12, a novel CXCL12 inhibitor, interferes with chronic lymphocytic leukemia cell motility and causes chemosensitization. Blood.

[145] Sczepanski J.T., Joyce G.F. (2013). Binding of a structured D-RNA molecule by an L-RNA aptamer. J. Am. Chem. Soc..

[146] Vater A., Sell S., Kaczmarek P., Maasch C., Buchner K., Pruszynska-Oszmalek E., Kolodziejski P., Purschke W.G., Nowak K.W., Strowski M.Z. (2013). A mixed mirror-image DNA/RNA aptamer inhibits glucagon and acutely improves glucose tolerance in models of type 1 and type 2 diabetes. J. Biol. Chem..

[147] Wlotzka B., Leva S., Eschgfaller B., Burmeister J., Kleinjung F., Kaduk C., Muhn P., Hess-Stumpp H., Klussmann S. (2002). In Vivo properties of an anti-GnRH Spiegelmer: An example of an oligonucleotide-based therapeutic substance class. Proc. Natl. Acad. Sci. USA.

[148] Leva S., Lichte A., Burmeister J., Muhn P., Jahnke B., Fesser D., Erfurth J., Burgstaller P., Klussmann S. (2002). GnRH Binding RNA and DNA Spiegelmers: A Novel Approach toward GnRH Antagonism. Chem. Biol..

[149] Purschke W.G., Radtke F., Kleinjung F., Klussmann S. (2003). A DNA Spiegelmer to staphylococcal enterotoxin B. Nucleic Acids Res..

[150] Kabza A.M., Sczepanski J.T. (2017). An l-RNA Aptamer with Expanded Chemical Functionality that Inhibits MicroRNA Biogenesis. ChemBioChem.

[151] Taylor A.I., Holliger P. (2018). Selecting Fully-Modified XNA Aptamers Using Synthetic Genetics. Curr. Protoc. Chem. Biol..

[152] Biondi E., Benner S.A. (2018). Artificially Expanded Genetic Information Systems for New Aptamer Technologies. Biomedicines.

[153] Hernandez A.R., Shao Y., Hoshika S., Yang Z., Shelke S.A., Herrou J., Kim H.-J., Kim M.-J., Piccirilli J.A., Benner S.A. (2015). A Crystal Structure of a Functional RNA Molecule Containing an Artificial Nucleobase Pair. Angew. Chem..

[154] Biondi E., Lane J.D., Das D., Dasgupta S., Piccirilli J.A., Hoshika S., Bradley K.M., Krantz B.A., Benner S.A. (2016). Laboratory evolution of artificially expanded DNA gives redesignable aptamers that target the toxic form of anthrax protective antigen. Nucleic Acids Res..

[155] Di Giusto D.A., King G.C. (2004). Construction, stability, and activity of multivalent circular anticoagulant aptamers. J. Biol. Chem..

[156] Kuai H., Zhao Z., Mo L., Liu H., Hu X., Fu T., Zhang X., Tan W. (2017). Circular Bivalent Aptamers Enable in vivo Stability and Recognition. J. Am. Chem. Soc..

[157] Riccardi C., Meyer A., Vasseur J.-J., Russo Krauss I., Paduano L., Morvan F., Montesarchio D. (2019). Fine-tuning the properties of the thrombin binding aptamer through cyclization: Effect of the 5′-3′ connecting linker on the aptamer stability and anticoagulant activity. Bioorg. Chem..

[158] Riccardi C., Meyer A., Vasseur J.-J., Russo Krauss I., Paduano L., Oliva R., Petraccone L., Morvan F., Montesarchio D. (2019). Stability Is Not Everything: The Case of the Cyclisation of a Thrombin-Binding Aptamer. ChemBioChem.

[159] Shi S.H., Hayashi Y., Petralia R.S., Zaman S.H., Wenthold R.J., Svoboda K., Malinow R. (1999). Rapid spine delivery and redistribution of AMPA receptors after synaptic NMDA receptor activation. Science.

[160] Vorobyeva M., Vorobjev P., Venyaminova A. (2016). Multivalent Aptamers: Versatile Tools for Diagnostic and Therapeutic Applications. Molecules.

[161] Hasegawa H., Savory N., Abe K., Ikebukuro K. (2016). Methods for Improving Aptamer Binding Affinity. Molecules.

[162] Hasegawa H., Sode K., Ikebukuro K. (2008). Selection of DNA aptamers against VEGF165 using a protein competitor and the aptamer blotting method. Biotechnol. Lett..

[163] Batool S., Argyropoulos K.V., Azad R., Okeoma P., Zumrut H., Bhandari S., Dekhang R., Mallikaratchy P.R. (2019). Dimerization of an aptamer generated from Ligand-guided selection (LIGS) yields a high affinity scaffold against B-cells. Biochim. Biophys. Acta Gen. Subj..

[164] Wu Y., Sefah K., Liu H., Wang R., Tan W. (2010). DNA aptamer-micelle as an efficient detection/delivery vehicle toward cancer cells. Proc. Natl. Acad. Sci. USA.

[165] Xing H., Tang L., Yang X., Hwang K., Wang W., Yin Q., Wong N.Y., Dobrucki L.W., Yasui N., Katzenellenbogen J.A. (2013). Selective Delivery of an Anticancer Drug with Aptamer-Functionalized Liposomes to Breast Cancer Cells in vitro and in Vivo. J. Mater. Chem. B.

[166] Cao Z., Tong R., Mishra A., Xu W., Wong G.C., Cheng J., Lu Y. (2009). Reversible cell-specific drug delivery with aptamer-functionalized liposomes. Angew. Chem. Int. Ed. Engl..

[167] Riccardi C., Fàbrega C., Grijalvo S., Vitiello G., D’Errico G., Eritja R., Montesarchio D. (2018). AS1411-decorated niosomes as effective nanocarriers for Ru(iii)-based drugs in anticancer strategies. J. Mater. Chem. B.

[168] Li Z., Liu Z., Yin M., Yang X., Yuan Q., Ren J., Qu X. (2012). Aptamer-capped multifunctional mesoporous strontium hydroxyapatite nanovehicle for cancer-cell-responsive drug delivery and imaging. Biomacromolecules.

[169] Cammarata C.R., Hughes M.E., Ofner C.M. (2015). Carbodiimide induced cross-linking, ligand addition, and degradation in gelatin. Mol. Pharm..

[170] Mann A.P., Bhavane R.C., Somasunderam A., Liz Montalvo-Ortiz B., Ghaghada K.B., Volk D., Nieves-Alicea R., Suh K.S., Ferrari M., Annapragada A. (2011). Thioaptamer conjugated liposomes for tumor vasculature targeting. Oncotarget.

[171] Zhang K., Liu M., Tong X., Sun N., Zhou L., Cao Y., Wang J., Zhang H., Pei R. (2015). Aptamer-Modified Temperature-Sensitive Liposomal Contrast Agent for Magnetic Resonance Imaging. Biomacromolecules.

[172] Zhang J., Chen R., Fang X., Chen F., Wang Y., Chen M. (2015). Nucleolin targeting AS1411 aptamer modified pH-sensitive micelles for enhanced delivery and antitumor efficacy of paclitaxel. Nano Res..

[173] Li X., Yu Y., Ji Q., Qiu L. (2015). Targeted delivery of anticancer drugs by aptamer AS1411 mediated Pluronic F127/cyclodextrin-linked polymer composite micelles. Nanomedicine.

[174] Xu W., Siddiqui I.A., Nihal M., Pilla S., Rosenthal K., Mukhtar H., Gong S. (2013). Aptamer-conjugated and doxorubicin-loaded unimolecular micelles for targeted therapy of prostate cancer. Biomaterials.

[175] Prabhu H.R., Patravale V.B., Joshi M.D. (2015). Polymeric nanoparticles for targeted treatment in oncology: Current insights. Int. J. Nanomed..

[176] Cheng J., Teply B.A., Sherifi I., Sung J., Luther G., Gu F.X., Levy-Nissenbaum E., Radovic-Moreno A.F., Langer R., Farokhzad O.C. (2007). Formulation of functionalized PLGA-PEG nanoparticles for in vivo targeted drug delivery. Biomaterials.

[177] Tong R., Yala L., Fan T.M., Cheng J. (2010). The formulation of aptamer-coated paclitaxel-polylactide nanoconjugates and their targeting to cancer cells. Biomaterials.

[178] Farokhzad O.C., Cheng J., Teply B.A., Sherifi I., Jon S., Kantoff P.W., Richie J.P., Langer R. (2006). Targeted nanoparticle-aptamer bioconjugates for cancer chemotherapy in vivo. Proc. Natl. Acad. Sci. USA.

[179] Guo J., Gao X., Su L., Xia H., Gu G., Pang Z., Jiang X., Yao L., Chen J., Chen H. (2011). Aptamer-functionalized PEG-PLGA nanoparticles for enhanced anti-glioma drug delivery. Biomaterials.

[180] Yu C., Hu Y., Duan J., Yuan W., Wang C., Xu H., Yang X.D. (2011). Novel aptamer-nanoparticle bioconjugates enhances delivery of anticancer drug to MUC1-positive cancer cells in vitro. PLoS ONE.

[181] Das M., Duan W., Sahoo S.K. (2015). Multifunctional nanoparticle-EpCAM aptamer bioconjugates: A paradigm for targeted drug delivery and imaging in cancer therapy. Nanomedicine.

[182] Alibolandi M., Ramezani M., Sadeghi F., Abnous K., Hadizadeh F. (2015). Epithelial cell adhesion molecule aptamer conjugated PEG-PLGA nanopolymersomes for targeted delivery of doxorubicin to human breast adenocarcinoma cell line in vitro. Int. J. Pharm..

[183] Ni M., Xiong M., Zhang X., Cai G., Chen H., Zeng Q., Yu Z. (2015). Poly(lactic-co-glycolic acid) nanoparticles conjugated with CD133 aptamers for targeted salinomycin delivery to CD133+ osteosarcoma cancer stem cells. Int. J. Nanomed..

[184] Aravind A., Jeyamohan P., Nair R., Veeranarayanan S., Nagaoka Y., Yoshida Y., Maekawa T., Kumar D.S. (2012). AS1411 aptamer tagged PLGA-lecithin-PEG nanoparticles for tumor cell targeting and drug delivery. Biotechnol. Bioeng..

[185] Ghasemi Z., Dinarvand R., Mottaghitalab F., Esfandyari-Manesh M., Sayari E., Atyabi F. (2015). Aptamer decorated hyaluronan/chitosan nanoparticles for targeted delivery of 5-fluorouracil to MUC1 overexpressing adenocarcinomas. Carbohydr. Polym..

[186] Sayari E., Dinarvand M., Amini M., Azhdarzadeh M., Mollarazi E., Ghasemi Z., Atyabi F. (2014). MUC1 aptamer conjugated to chitosan nanoparticles, an efficient targeted carrier designed for anticancer SN38 delivery. Int. J. Pharm..

[187] Li J., You J., Dai Y., Shi M., Han C., Xu K. (2014). Gadolinium oxide nanoparticles and aptamer-functionalized silver nanoclusters-based multimodal molecular imaging nanoprobe for optical/magnetic resonance cancer cell imaging. Anal. Chem..

[188] Bagalkot V., Zhang L., Levy-Nissenbaum E., Jon S., Kantoff P.W., Langer R., Farokhzad O.C. (2007). Quantum dot-aptamer conjugates for synchronous cancer imaging, therapy, and sensing of drug delivery based on bi-fluorescence resonance energy transfer. Nano Lett..

[189] Cai L., Chen Z.Z., Chen M.Y., Tang H.W., Pang D.W. (2013). MUC-1 aptamer-conjugated dye-doped silica nanoparticles for MCF-7 cells detection. Biomaterials.

[190] Zhang H. (2004). Thermally cross-linked superparamagnetic iron oxide nanoparticle-A10 RNA aptamer-doxorubicin conjugate. Molecular Imaging and Contrast Agent Database (MICAD).

[191] Deng K., Hou Z., Li X., Li C., Zhang Y., Deng X. (2015). Aptamer-Mediated Up-conversion Core/MOF Shell Nanocomposites for Targeted Drug Delivery and Cell Imaging. Sci. Rep..

[192] Yang X., Liu X., Liu Z., Pu F., Ren J., Qu X. (2012). Near-Infrared Light-Triggered, Targeted Drug Delivery to Cancer Cells by Aptamer Gated Nanovehicles. Adv. Mater..

[193] Chen T., Shukoor M.I., Wang R., Zhao Z., Yuan Q., Bamrungsap S., Xiong X., Tan W. (2011). Smart Multifunctional Nanostructure for Targeted Cancer Chemotherapy and Magnetic Resonance Imaging. ACS Nano.

[194] Kang H., O’Donoghue M.B., Liu H., Tan W. (2010). A liposome-based nanostructure for aptamer directed delivery. Chem. Commun..

[195] Ara M.N., Matsuda T., Hyodo M., Sakurai Y., Hatakeyama H., Ohga N., Hida K., Harashima H. (2014). An aptamer ligand based liposomal nanocarrier system that targets tumor endothelial cells. Biomaterials.

[196] Li L., Hou J., Liu X., Guo Y., Wu Y., Zhang L., Yang Z. (2014). Nucleolin-targeting liposomes guided by aptamer AS1411 for the delivery of siRNA for the treatment of malignant melanomas. Biomaterials.

[197] Ababneh N., Alshaer W., Allozi O., Mahafzah A., El-Khateeb M., Hillaireau H., Noiray M., Fattal E., Ismail S. (2013). In Vitro selection of modified RNA aptamers against CD44 cancer stem cell marker. Nucleic Acid Ther..

[198] Alshaer W., Hillaireau H., Vergnaud J., Ismail S., Fattal E. (2015). Functionalizing Liposomes with anti-CD44 Aptamer for Selective Targeting of Cancer Cells. Bioconjug. Chem..

[199] Alshaer W., Hillaireau H., Vergnaud J., Mura S., Delomenie C., Sauvage F., Ismail S., Fattal E. (2018). Aptamer-guided siRNA-loaded nanomedicines for systemic gene silencing in CD-44 expressing murine triple-negative breast cancer model. J. Control. Release.

[200] Liao Z.X., Chuang E.Y., Lin C.C., Ho Y.C., Lin K.J., Cheng P.Y., Chen K.J., Wei H.J., Sung H.W. (2015). An AS1411 aptamer-conjugated liposomal system containing a bubble-generating agent for tumor-specific chemotherapy that overcomes multidrug resistance. J. Control. Release.

[201] Yu S., Dong R., Chen J., Chen F., Jiang W., Zhou Y., Zhu X., Yan D. (2014). Synthesis and self-assembly of amphiphilic aptamer-functionalized hyperbranched multiarm copolymers for targeted cancer imaging. Biomacromolecules.

[202] Wu X., Ding B., Gao J., Wang H., Fan W., Wang X., Zhang W., Wang X., Ye L., Zhang M. (2011). Second-generation aptamer-conjugated PSMA-targeted delivery system for prostate cancer therapy. Int. J. Nanomed..

[203] Pilapong C., Sitthichai S., Thongtem S., Thongtem T. (2014). Smart magnetic nanoparticle-aptamer probe for targeted imaging and treatment of hepatocellular carcinoma. Int. J. Pharm..

[204] Kishore V., Paderi J.E., Akkus A., Smith K.M., Balachandran D., Beaudoin S., Panitch A., Akkus O. (2011). Incorporation of a decorin biomimetic enhances the mechanical properties of electrochemically aligned collagen threads. Acta Biomater..

[205] Pala K., Serwotka A., Jelen F., Jakimowicz P., Otlewski J. (2014). Tumor-specific hyperthermia with aptamer-tagged superparamagnetic nanoparticles. Int. J. Nanomed..

[206] Wang K., Yao H., Meng Y., Wang Y., Yan X., Huang R. (2015). Specific aptamer-conjugated mesoporous silica-carbon nanoparticles for HER2-targeted chemo-photothermal combined therapy. Acta Biomater..

[207] Baek S.E., Lee K.H., Park Y.S., Oh D.K., Oh S., Kim K.S., Kim D.E. (2014). RNA aptamer-conjugated liposome as an efficient anticancer drug delivery vehicle targeting cancer cells in vivo. J. Control. Release.

[208] Charoenphol P., Bermudez H. (2014). Aptamer-Targeted DNA Nanostructures for Therapeutic Delivery. Mol. Pharm..

[209] Chang M., Yang C.S., Huang D.M. (2011). Aptamer-conjugated DNA icosahedral nanoparticles as a carrier of doxorubicin for cancer therapy. ACS Nano.

[210] Kurosaki T., Higuchi N., Kawakami S., Higuchi Y., Nakamura T., Kitahara T., Hashida M., Sasaki H. (2012). Self-assemble gene delivery system for molecular targeting using nucleic acid aptamer. Gene.

[211] Zhao N., Bagaria H.G., Wong M.S., Zu Y. (2011). A nanocomplex that is both tumor cell-selective and cancer gene-specific for anaplastic large cell lymphoma. J. Nanobiotechnol..

[212] Subramanian N., Kanwar J.R., Athalya P.K., Janakiraman N., Khetan V., Kanwar R.K., Eluchuri S., Krishnakumar S. (2015). EpCAM aptamer mediated cancer cell specific delivery of EpCAM siRNA using polymeric nanocomplex. J. Biomed. Sci..

[213] Lee I.H., An S., Yu M.K., Kwon H.K., Im S.H., Jon S. (2011). Targeted chemoimmunotherapy using drug-loaded aptamer-dendrimer bioconjugates. J. Control. Release.

[214] Zhang H., Ma Y., Xie Y., An Y., Huang Y., Zhu Z., Yang C.J. (2015). A controllable aptamer-based self-assembled DNA dendrimer for high affinity targeting, bioimaging and drug delivery. Sci. Rep..

[215] Green N. (1963). Avidin. 1. The USE of [^14^C] Biotin for Kinetic Studies and for Assay. Biochem. J..

[216] Zhou C., Chen T., Wu C., Zhu G., Qiu L., Cui C., Hou W., Tan W. (2015). Aptamer CaCO_3_ nanostructures: A facile, pH-responsive, specific platform for targeted anticancer theranostics. Chem. Asian J..

[217] Yu Y., Duan S., He J., Liang W., Su J., Zhu J., Hu N., Zhao Y., Lu X. (2016). Highly sensitive detection of leukemia cells based on aptamer and quantum dots. Oncol. Rep..

[218] Xue Y., Li X., Li H., Zhang W. (2014). Quantifying thiol–gold interactions towards the efficient strength control. Nat. Commun..

[219] Zhang X., Servos M.R., Liu J. (2012). Instantaneous and Quantitative Functionalization of Gold Nanoparticles with Thiolated DNA Using a pH-Assisted and Surfactant-Free Route. J. Am. Chem. Soc..

[220] Dam H.D., Culver K.S., Odom T.W. (2014). Grafting aptamers onto gold nanostars increases in vitro efficacy in a wide range of cancer cell types. Mol. Pharm..

[221] Shiao Y.S., Chiu H.H., Wu P.H., Huang Y.F. (2014). Aptamer-functionalized gold nanoparticles as photoresponsive nanoplatform for co-drug delivery. ACS Appl. Mater. Interfaces.

[222] Luo L.Y., Shiao Y.S., Huang Y.F. (2011). Release of photoactivatable drugs from plasmonic nanoparticles for targeted cancer therapy. ACS Nano.

[223] Wang J., Zhu G., You M., Song E., Shukoor M.I., Zhang K., Altman M.B., Chen Y., Zhu Z., Huang C.Z. (2012). Assembly of aptamer switch probes and photosensitizer on gold nanorods for targeted photothermal and photodynamic cancer therapy. ACS Nano.

[224] Huang Y.F., Sefah K., Bamrungsap S., Chang H.T., Tan W. (2008). Selective photothermal therapy for mixed cancer cells using aptamer-conjugated nanorods. Langmuir.

[225] Yang L., Tseng Y.T., Suo G., Chen L., Yu J., Chiu W.J., Huang C.C., Lin C.H. (2015). Photothermal therapeutic response of cancer cells to aptamer-gold nanoparticle-hybridized graphene oxide under NIR illumination. ACS Appl. Mater. Interfaces.

[226] Zhao F., Zhou J., Su X., Wang Y., Yan X., Jia S., Du B. (2017). A Smart Responsive Dual Aptamers-Targeted Bubble-Generating Nanosystem for Cancer Triplex Therapy and Ultrasound Imaging. Small.

[227] Hooker M.J., Esser-Kahn A., Francis M.B. (2006). Modification of aniline containing proteins using an oxidative coupling strategy. J. Am. Chem. Soc..

[228] Mastico A.R., Talbot S.J., Stockley P.G. (1993). Multiple presentation of foreign peptides on the surface of an RNA-free spherical bacteriophage capsid. J. Gen. Virol..

[229] Carrico Z.M., Romanini D.W., Mehl R.A., Francis M.B. (2008). Oxidative coupling of peptides to a virus capsid containing unnatural amino acids. Chem. Commun..

[230] Oh S.S., Lee B.F., Leibfarth F.A., Eisenstein M., Robb M.J., Lynd N.A., Hawker C.J., Soh H.T. (2014). Synthetic Aptamer-Polymer Hybrid Constructs for Programmed Drug Delivery into Specific Target Cells. J. Am. Chem. Soc..

[231] Aravind A., Varghese S.H., Veeranarayanan S., Mathew A., Nagaoka Y., Iwai S., Fukuda T., Hasumura T., Yoshida Y., Maekawa T. (2012). Aptamer-labeled PLGA nanoparticles for targeting cancer cells. Cancer Nanotechnol..

[232] Li L.L., Yin Q., Cheng J., Lu Y. (2012). Polyvalent mesoporous silica nanoparticle-aptamer bioconjugates target breast cancer cells. Adv. Healthc. Mater..

[233] Alibolandi M., Taghdisi S.M., Ramezani P., Hosseini Shamili F., Farzad S.A., Abnous K., Ramezani M. (2017). Smart AS1411-aptamer conjugated pegylated PAMAM dendrimer for the superior delivery of camptothecin to colon adenocarcinoma in vitro and in vivo. Int. J. Pharm..

[234] Tao W., Zeng X., Wu J., Zhu X., Yu X., Zhang X., Zhang J., Liu G., Mei L. (2016). Polydopamine-Based Surface Modification of Novel Nanoparticle-Aptamer Bioconjugates for in vivo Breast Cancer Targeting and Enhanced Therapeutic Effects. Theranostics.

[235] Mosafer J., Teymouri M., Abnous K., Tafaghodi M., Ramezani M. (2017). Study and evaluation of nucleolin-targeted delivery of magnetic PLGA-PEG nanospheres loaded with doxorubicin to C6 glioma cells compared with low nucleolin-expressing L929 cells. Mater. Sci. Eng. C Mater. Biol. Appl..

[236] Li X., Zhu X., Qiu L. (2016). Constructing aptamer anchored nanovesicles for enhanced tumor penetration and cellular uptake of water soluble chemotherapeutics. Acta Biomater..

[237] Barzegar Behrooz A., Nabavizadeh F., Adiban J., Shafiee Ardestani M., Vahabpour R., Aghasadeghi M.R., Sohanaki H. (2017). Smart bomb AS1411 aptamer-functionalized/PAMAM dendrimer nanocarriers for targeted drug delivery in the treatment of gastric cancer. Clin. Exp. Pharmacol. Physiol..

[238] Ayatollahi S., Salmasi Z., Hashemi M., Askarian S., Oskuee R.K., Abnous K., Ramezani M. (2017). Aptamer-targeted delivery of Bcl-xL shRNA using alkyl modified PAMAM dendrimers into lung cancer cells. Int. J. Biochem. Cell Biol..

[239] Bandekar A., Zhu C., Jindal R., Bruchertseifer F., Morgenstern A., Sofou S. (2014). Anti-prostate-specific membrane antigen liposomes loaded with 225Ac for potential targeted antivascular alpha-particle therapy of cancer. J. Nucl. Med..

[240] Dhar S., Gu F.X., Langer R., Farokhzad O.C., Lippard S.J. (2008). Targeted delivery of cisplatin to prostate cancer cells by aptamer functionalized Pt(IV) prodrug-PLGA-PEG nanoparticles. Proc. Natl. Acad. Sci. USA.

[241] Yu M.K., Kim D., Lee I.H., So J.S., Jeong Y.Y., Jon S. (2011). Image-guided prostate cancer therapy using aptamer-functionalized thermally cross-linked superparamagnetic iron oxide nanoparticles. Small.

[242] Leach J.C., Wang A., Ye K., Jin S. (2016). A RNA-DNA Hybrid Aptamer for Nanoparticle-Based Prostate Tumor Targeted Drug Delivery. Int. J. Mol. Sci..

[243] Hao Z., Fan W., Hao J., Wu X., Zeng G.Q., Zhang L.J., Nie S.F., Wang X.D. (2016). Efficient delivery of micro RNA to bone-metastatic prostate tumors by using aptamer-conjugated atelocollagen in vitro and in vivo. Drug Deliv..

[244] Lin Z., Ma Q., Fei X., Zhang H., Su X. (2014). A novel aptamer functionalized CuInS2 quantum dots probe for daunorubicin sensing and near infrared imaging of prostate cancer cells. Anal. Chim. Acta.

[245] Zhang C., Ji X., Zhang Y., Zhou G., Ke X., Wang H., Tinnefeld P., He Z. (2013). One-pot synthesized aptamer-functionalized CdTe:Zn^2+^ quantum dots for tumor-targeted fluorescence imaging in vitro and in vivo. Anal. Chem..

[246] Guo F., Hu Y., Yu L., Deng X., Meng J., Wang C., Yang X.D. (2016). Enhancement of Thermal Damage to Adenocarcinoma Cells by Iron Nanoparticles Modified with MUC1 Aptamer. J. Nanosci. Nanotechnol..

[247] Esfandyari-Manesh M., Mohammadi A., Atyabi F., Nabavi S.M., Ebrahimi S.M., Shahmoradi E., Varnamkhasti B.S., Ghahremani M.H., Dinarvand R. (2016). Specific targeting delivery to MUC1 overexpressing tumors by albumin-chitosan nanoparticles conjugated to DNA aptamer. Int. J. Pharm..

[248] Perepelyuk M., Maher C., Lakshmikuttyamma A., Shoyele S.A. (2016). Aptamer-hybrid nanoparticle bioconjugate efficiently delivers miRNA-29b to non-small-cell lung cancer cells and inhibits growth by downregulating essential oncoproteins. Int. J. Nanomed..

[249] Azhdarzadeh M., Atyabi F., Saei A.A., Varnamkhasti B.S., Omidi Y., Fateh M., Ghavami M., Shanehsazzadeh S., Dinarvand R. (2016). Theranostic MUC-1 aptamer targeted gold coated superparamagnetic iron oxide nanoparticles for magnetic resonance imaging and photothermal therapy of colon cancer. Colloids Surf. B Biointerfaces.

[250] Charbgoo F., Alibolandi M., Taghdisi S.M., Abnous K., Soltani F., Ramezani M. (2018). MUC1 aptamer-targeted DNA micelles for dual tumor therapy using doxorubicin and KLA peptide. Nanomedicine.

[251] Taghavi S., Ramezani M., Alibolandi M., Abnous K., Taghdisi S.M. (2017). Chitosan-modified PLGA nanoparticles tagged with 5TR1 aptamer for in vivo tumor-targeted drug delivery. Cancer Lett..

[252] Xu C., Han X., Jiang Y., Yuan S., Wu Z., Wu Z., Qi X. (2017). Microenvironmental Control of MUC1 Aptamer-Guided Acid-Labile Nanoconjugate within Injectable Microporous Hydrogels. Bioconjug. Chem..

[253] Zhang Z., Ali M.M., Eckert M.A., Kang D.K., Chen Y.Y., Sender L.S., Fruman D.A., Zhao W. (2013). A polyvalent aptamer system for targeted drug delivery. Biomaterials.

[254] Huang F.Y., Chang H.-T., Tan W. (2008). Cancer Cell Targeting Using Multiple Aptamers Conjugated on Nanorods. Anal. Chem..

[255] Taghdisi S.M., Lavaee P., Ramezani M., Abnous K. (2011). Reversible targeting and controlled release delivery of daunorubicin to cancer cells by aptamer-wrapped carbon nanotubes. Eur. J. Pharm. Biopharm..

[256] Li J., Wu S., Wu C., Qiu L., Zhu G., Cui C., Liu Y., Hou W., Wang Y., Zhang L. (2016). Versatile surface engineering of porous nanomaterials with bioinspired polyphenol coatings for targeted and controlled drug delivery. Nanoscale.

[257] Taghdisi S.M., Danesh N.M., Lavaee P., Emrani A.S., Hassanabad K.Y., Ramezani M., Abnous K. (2016). Double targeting, controlled release and reversible delivery of daunorubicin to cancer cells by polyvalent aptamers-modified gold nanoparticles. Mater. Sci. Eng. C Mater. Biol. Appl..

[258] Kang S., Luo Y.L., Huang Y.F., Yeh C.K. DNA-conjugated gold nanoparticles for ultrasound targeted drug delivery. Proceedings of the 2012 IEEE International Ultrasonics Symposium.

[259] Wang C.H., Kang S.T., Lee Y.H., Luo Y.L., Huang Y.F., Yeh C.K. (2012). Aptamer-conjugated and drug-loaded acoustic droplets for ultrasound theranosis. Biomaterials.

[260] Zhou J., Soontornworajit B., Wang Y. (2010). A temperature-responsive antibody-like nanostructure. Biomacromolecules.

[261] Fan Z., Shelton M., Singh A.K., Senapati D., Khan S.A., Ray P.C. (2012). Multifunctional plasmonic shell-magnetic core nanoparticles for targeted diagnostics, isolation, and photothermal destruction of tumor cells. ACS Nano.

[262] Moosavian S.A., Abnous K., Badiee A., Jaafari M.R. (2016). Improvement in the drug delivery and anti-tumor efficacy of PEGylated liposomal doxorubicin by targeting RNA aptamers in mice bearing breast tumor model. Colloids Surf. B Biointerfaces.

[263] Powell D., Chandra S., Dodson K., Shaheen F., Wiltz K., Ireland S., Syed M., Dash S., Wiese T., Mandal T. (2017). Aptamer-functionalized hybrid nanoparticle for the treatment of breast cancer. Eur. J. Pharm. Biopharm..

[264] Fan W., Wang X., Ding B., Cai H., Wang X., Fan Y., Li Y., Liu S., Nie S., Lu Q. (2016). Thioaptamer-conjugated CD44-targeted delivery system for the treatment of breast cancer in vitro and in vivo. J. Drug Target..

[265] Li L., Xiang D., Shigdar S., Yang W., Li Q., Lin J., Liu K., Duan W. (2014). Epithelial cell adhesion molecule aptamer functionalized PLGA-lecithin-curcumin-PEG nanoparticles for targeted drug delivery to human colorectal adenocarcinoma cells. Int. J. Nanomed..

[266] Xie X., Li F., Zhang H., Lu Y., Lian S., Lin H., Gao Y., Jia L. (2016). EpCAM aptamer-functionalized mesoporous silica nanoparticles for efficient colon cancer cell-targeted drug delivery. Eur. J. Pharm. Sci..

[267] Yu Z., Chen F., Qi X., Dong Y., Zhang Y., Ge Z., Cai G., Zhang X. (2018). Epidermal growth factor receptor aptamer-conjugated polymer-lipid hybrid nanoparticles enhance salinomycin delivery to osteosarcoma and cancer stem cells. Exp. Ther. Med..

[268] Gu M.J., Li K.F., Zhang L.X., Wang H., Liu L.S., Zheng Z.Z., Han N.Y., Yang Z.J., Fan T.Y. (2015). In Vitro study of novel gadolinium-loaded liposomes guided by GBI-10 aptamer for promising tumor targeting and tumor diagnosis by magnetic resonance imaging. Int. J. Nanomed..

[269] Chen X.C., Deng Y.L., Lin Y., Pang D.W., Qing H., Qu F., Xie H.Y. (2008). Quantum dot-labeled aptamer nanoprobes specifically targeting glioma cells. Nanotechnology.

[270] Monaco I., Camorani S., Colecchia D., Locatelli E., Calandro P., Oudin A., Niclou S., Arra C., Chiariello M., Cerchia L. (2017). Aptamer Functionalization of Nanosystems for Glioblastoma Targeting through the Blood-Brain Barrier. J. Med. Chem..

[271] Song X., Ren Y., Zhang J., Wang G., Han X., Zheng W., Zhen L. (2015). Targeted delivery of doxorubicin to breast cancer cells by aptamer functionalized DOTAP/DOPE liposomes. Oncol. Rep..

[272] Belyanina I.V., Zamay T.N., Zamay G.S., Zamay S.S., Kolovskaya O.S., Ivanchenko T.I., Denisenko V.V., Kirichenko A.K., Glazyrin Y.E., Garanzha I.V. (2017). In Vivo Cancer Cells Elimination Guided by Aptamer-Functionalized Gold-Coated Magnetic Nanoparticles and Controlled with Low Frequency Alternating Magnetic Field. Theranostics.

[273] Chandrasekaran R., Lee A.S., Yap L.W., Jans D.A., Wagstaff K.M., Cheng W. (2016). Tumor cell-specific photothermal killing by SELEX-derived DNA aptamer-targeted gold nanorods. Nanoscale.

[274] Jurek P.M., Zablocki K., Wasko U., Mazurek M.P., Otlewski J., Jelen F. (2017). Anti-FGFR1 aptamer-tagged superparamagnetic conjugates for anticancer hyperthermia therapy. Int. J. Nanomed..

[275] Taghdisi S.M., Danesh N.M., Ramezani M., Lavaee P., Jalalian S.H., Robati R.Y., Abnous K. (2016). Double targeting and aptamer-assisted controlled release delivery of epirubicin to cancer cells by aptamers-based dendrimer in vitro and in vivo. Eur. J. Pharm. Biopharm..

[276] Pi F., Zhang H., Li H. (2017). RNA nanoparticles harboring annexin A2 aptamer can target ovarian cancer for tumor-specific doxorubicin delivery. Nanomedicine.

[277] Zeng Y.B., Yu Z.C., He Y.N., Zhang T., Du L.B., Dong Y.M., Chen H.W., Zhang Y.Y., Wang W.Q. (2018). Salinomycin-loaded lipid-polymer nanoparticles with anti-CD20 aptamers selectively suppress human CD20+ melanoma stem cells. Acta Pharmacol. Sin..

[278] Eyetech Study Group (2003). Anti-vascular endothelial growth factor therapy for subfoveal choroidal neovascularization secondary to age-related macular degeneration: Phase II study results. Ophthalmology.

[279] Retina E.S.G.J. (2002). Preclinical and phase 1A clinical evaluation of an anti-VEGF pegylated aptamer (EYE001) for the treatment of exudative age-related macular degeneration. Retina.

[280] Drolet D.W., Nelson J., Tucker C.E., Zack P.M., Nixon K., Bolin R., Judkins M.B., Farmer J.A., Wolf J.L., Gill S.C. (2000). Pharmacokinetics and safety of an anti-vascular endothelial growth factor aptamer (NX1838) following injection into the vitreous humor of rhesus monkeys. Pharm. Res..

[281] Ismail S.I., Alshaer W. (2018). Therapeutic aptamers in discovery, preclinical and clinical stages. Adv. Drug Deliv. Rev..

[282] Hassel S.K., Mayer G. (2019). Aptamers as Therapeutic Agents: Has the Initial Euphoria Subsided?. Mol. Diagn. Ther..

[283] D’Amico D.J. (2006). Pegaptanib Sodium for Neovascular Age-Related Macular Degeneration: Two-Year Safety Results of the Two Prospective, Multicenter, Controlled Clinical Trials. Ophthalmology.

[284] Goebl N., Berridge B., Wroblewski V.J., Brown-Augsburger P.L. (2007). Development of a Sensitive and Specific in Situ Hybridization Technique for the Cellular Localization of Antisense Oligodeoxynucleotide Drugs in Tissue Sections. Toxicol. Pathol..

[285] Farman C.A., Kornbrust D.J. (2003). Oligodeoxynucleotide Studies in Primates: Antisense and Immune Stimulatory Indications. Toxicol. Pathol..

[286] Henry S.P., Giclas P.C., Leeds J., Pangburn M., Auletta C., Levin A.A., Kornbrust D.J. (1997). Activation of the alternative pathway of complement by a phosphorothioate oligonucleotide: Potential mechanism of action. J. Pharmacol. Exp. Ther..

[287] Avci-Adali M., Steinle H., Michel T., Schlensak C., Wendel H.P. (2013). Potential Capacity of Aptamers to Trigger Immune Activation in Human Blood. PLoS ONE.

[288] Bruno J.G. (2018). Potential Inherent Stimulation of the Innate Immune System by Nucleic Acid Aptamers and Possible Corrective Approaches. Pharmaceuticals.

[289] Swayze E.E., Siwkowski A.M., Wancewicz E.V., Migawa M.T., Wyrzykiewicz T.K., Hung G., Monia B.P., Bennett A.C.F. (2006). Antisense oligonucleotides containing locked nucleic acid improve potency but cause significant hepatotoxicity in animals. Nucleic Acids Res..

[290] Burdick A.D., Sciabola S., Mantena S.R., Hollingshead B.D., Stanton R., Warneke J.A., Zeng M., Martsen E., Medvedev A., Makarov S.S. (2014). Sequence motifs associated with hepatotoxicity of locked nucleic acid—modified antisense oligonucleotides. Nucleic Acids Res..

[291] Waring M.J. (2010). Lipophilicity in drug discovery. Expert Opin. Drug Discov..

[292] Lee Y., Urban J.H., Xu L., Sullenger B.A., Lee J. (2016). 2′Fluoro Modification Differentially Modulates the Ability of RNAs to Activate Pattern Recognition Receptors. Nucleic Acid Ther..

[293] Ganson N.J., Povsic T.J., Sullenger B.A., Alexander J.H., Zelenkofske S.L., Sailstad J.M., Rusconi C.P., Hershfield M.S. (2016). Pre-existing anti-polyethylene glycol antibody linked to first-exposure allergic reactions to pegnivacogin, a PEGylated RNA aptamer. J. Allergy Clin. Immunol..

